# Acute inhibition of OGA sex-dependently alters the networks associated with bioenergetics, autophagy, and neurodegeneration

**DOI:** 10.1186/s13041-022-00906-x

**Published:** 2022-03-05

**Authors:** Van N. Huynh, Gloria A. Benavides, Michelle S. Johnson, Xiaosen Ouyang, Balu K. Chacko, Edie Osuma, Toni Mueller, John Chatham, Victor M. Darley-Usmar, Jianhua Zhang

**Affiliations:** 1 Department of Pathology, Center for Free Radical Biology, University of Alabama at Birmingham, BMRII-534, 901 19th Street S., Birmingham, AL 35294-0017 USA; 2 Birmingham VA Medical Center, University of Alabama at Birmingham, Birmingham, AL 35294 USA

**Keywords:** Neurodegenerative disease, Autophagy, Mitochondria, O-GlcNAc, OGA, Cortex, Synapse, TCA cycle, Glycolysis

## Abstract

The accumulation of neurotoxic proteins characteristic of age-related neurodegenerative pathologies such as Alzheimer’s and Parkinson’s diseases is associated with the perturbation of metabolism, bioenergetics, and mitochondrial quality control. One approach to exploit these interactions therapeutically is to target the pathways that regulate metabolism. In this respect, the nutrient-sensing hexosamine biosynthesis pathway is of particular interest since it introduces a protein post-translational modification known as O-GlcNAcylation, which modifies different proteins in control versus neurodegenerative disease postmortem brains. A potent inhibitor of the O-GlcNAcase enzyme that removes the modification from proteins, Thiamet G (TG), has been proposed to have potential benefits in Alzheimer’s disease. We tested whether key factors in the O-GlcNAcylation are correlated with mitochondrial electron transport and proteins related to the autophagy/lysosomal pathways in the cortex of male and female mice with and without exposure to TG (10 mg/kg i.p.). Mitochondrial complex activities were measured in the protein homogenates, and a panel of metabolic, autophagy/lysosomal proteins and O-GlcNAcylation enzymes were assessed by either enzyme activity assay or by western blot analysis. We found that the networks associated with O-GlcNAcylation enzymes and activities with mitochondrial parameters, autophagy-related proteins as well as neurodegenerative disease-related proteins exhibited sex and TG dependent differences. Taken together, these studies provide a framework of interconnectivity for multiple O-GlcNAc-dependent pathways in mouse brain of relevance to aging and sex/age-dependent neurodegenerative pathogenesis and response to potential therapies.

**Supplementary Information:**

The online version contains supplementary material available at 10.1186/s13041-022-00906-x.

## Introduction

It is well established that the post-translational modification of proteins by O-linked β-N-acetylglucosamine (O-GlcNAc) contributes to the regulation of diverse cellular functions, including metabolism, mitochondrial function, autophagy, and cell survival [1, 2]. A number of studies have indicated that dysregulation of this pathway is a contributory factor to neurodegenerative diseases, and this pathway has been recognized as a candidate for therapeutic intervention [3–12]. For example, changes in protein O-GlcNAcylation levels have been reported in Alzheimer’s and Parkinson’s diseases [13–17]. In animal models, increasing O-GlcNAc levels by inhibition of O-GlcNAcase (OGA) increased tau O-GlcNAcylation, and decreased tau phosphorylation and associated neurodegenerative phenotypes [18–22]. Such observations encouraged further pre-clinical and clinical studies investigating the effects of pharmacological inhibition of OGA as a potential therapeutic strategy against neurodegenerative diseases [2, 23].

Since numerous proteins and cellular processes have been shown to be modulated by changes in O-GlcNAcylation, inhibition of OGA will likely have pleiotropic effects on neuronal function. Understanding these effects will be critical in the development of O-GlcNAc based therapies for neurodegenerative diseases. In primary neurons, we have shown that pharmacological inhibition of OGA in neurons increases O-GlcNAc levels, enhances MTOR phosphorylation, and suppresses autophagy, resulting in α-synuclein accumulation [16]. On the other hand, prolonged inhibition of OGA was reported to increase autophagy in an MTOR-independent manner in a neuron/glia mixed culture [24]. Increased autophagic flux has also been reported with TG administration in drinking water at 500 mg/kg/d for 2 weeks to RFP-GFP-LC3 mice [24]. In addition to apparently divergent effects on neuronal autophagy, an acute increase in O-GlcNAcylation levels in vivo impairs learning and memory [25] and it has been reported that protein O-GlcNAcylation is a regulator of neuronal excitability [25–27]. In other excitable cells, such as cardiomyocytes, increases in mitochondrial O-GlcNAc levels have been reported to increase oxidative phosphorylation as well as lead to impaired mitochondrial function [28–30].

The divergent effects of O-GlcNAcylation as a key mediator of neuronal function calls for an integrated view of its role in the regulation of autophagy, metabolism, and mitochondrial function in the brain. We propose that integrating bioenergetics with autophagy may reveal unique nodes of regulation, which can then serve as a novel focus for the development of therapeutics designed to improve both metabolic efficiency and decrease proteotoxicity. This approach requires statistical analysis methods to examine relationships between autophagy, bioenergetic and O-GlcNAcylation parameters within a cohort. We have recently shown how using such methods for the measurement of multiple inter-connected parameters in an integrative analysis can reveal the complex molecular interactions between pathways and disease mechanism [2, 3, 31–34].

To date, the majority of the studies related to the effects of increased O-GlcNAc in the brain have focused on chronic disease models or prolonged treatment with OGA inhibition, despite the fact that as discussed above short-term treatment with OGA inhibitors can have acute neurological effects [25–27]. We have recently shown that only 3 h after a single treatment with the OGA inhibitor thiamet G there was marked increases in O-GlcNAcylation (> 1.5-fold) of 65 proteins [35]. If chronic OGA inhibition is to be developed as a therapeutic tool for neurodegenerative diseases, it is critical that we develop a better understanding of the physiological responses to acute increases in O-GlcNAcylation. Therefore, in this study, using the same protocol as our prior proteomic studies, we examined the effects of 3 h of OGA inhibition with TG in male and female mouse brains. We measured a panel of metabolic, bioenergetic, mitochondrial, autophagic, neurodegeneration-related, and O-GlcNAc-related parameters. To determine the impact of protein O-GlcNAcylation on these parameters we performed Kendall’s bivariate analysis of the data for each group using data representing the autophagy, bioenergetic and O-GlcNAcylation pathways. We found a strong association between changes in mitochondrial and autophagy-related networks within some but not all of the groups. Of note was strong sex effect on the interactions between O-GlcNAcylation, mitochondrial function, and mitochondrial quality control.

## Methods

### Mice

All animal experiments were approved by the University of Alabama at Birmingham IACUC. C57BL/6 male mice were i.p. injected at 2 months of age with either saline or thiamet G (TG) at 10 mg/kg [35]. Cortex were dissected 3 h later and flash-frozen in liquid nitrogen. Cortex was used for bioenergetics, enzymatic, mitochondrial DNA (mtDNA), and western blot analyses (n = 12 male and n = 12 female for saline injection, and the same numbers of mice for TG). Immunohistochemistry was performed in 3 male mice each with saline versus TG at 10 mg/kg and half brain was fixed with 4% paraformaldehyde, dehydrated with sucrose, frozen in OCT before sectioning and immunostaining.

### Bioenergetic and mitochondrial assessment

Mitochondrial bioenergetics were assessed with frozen cortical tissues as described [36]. Cortex was pulverized in liquid nitrogen and then homogenized in 10 volumes of MAS buffer (70 mM Sucrose, 220 mM Mannitol, 5 mM KH_2_PO_4_, 5 mM MgCl_2_, 1 mM EGTA, 2 mM HEPES pH 7.4) with a glass-glass Dounce homogenizer. After centrifugation at 1000 × g for 10 min at 4 °C, the supernatant was collected and the protein concentration determined by DC Protein Assay (Bio-Rad). Equal amounts of protein were loaded onto Seahorse XF96 microplates (Agilent, Santa Clara, CA) and centrifuged at 2,000 × g for 20 min at 4 °C. Cytochrome C (10 µM) and Alamethicin (10 µg/ml) were then added. For complex I assays, 1 mM NADH was added. For complex II assays, 10 mM Succinate and 2 μM Rotenone were added. For complex III assays, 0.5 mM Duroquinol was added. For complex IV assays, 2 mM ascorbate and 0.5 mM TMPD were added. 2 µM Rotenone, 10 µM AA or 20 mM Azide were used to inhibit mitochondrial complexes.

### Mitochondrial citrate synthase activity assay

Citrate synthase and lactate dehydrogenase activities were measured using biochemical assays [36]. Protein lysates were incubated with oxaloacetate, acetyl CoA, and DTNB. The product of DTNB-CoA was measured at 412 nm, and the change of absorbance was used to calculate the activity as nmol/min/mg protein.

### LDHA activity assay

LDHA activity was measured as previously described [36]. Protein lysates were incubated in the presence of NADH and Pyruvate. The disappearance of NADH absorption at 340 nm was measured, and the change of absorbance was used to calculate the activity as nmol/min/mg protein.

### OGA activity assay

We measured the release of the fluorophore 4-methylumbelliferone (4MU) from 4MU-labeled GlcNAc and GalNAc substrates (Sigma-Aldrich) when incubated with the cortical lysates over time. Free 4MU (Sigma-Aldrich) in 100% EtOH was diluted to known concentrations (0.1, 0.5, 1, 5, or 10 nmol/well) to serve as standards. 4MU fluorescence was measured every 2 min for 20 min (excitation 368 nm, emission 450 nm). The change in 4MU fluorescence over time was used to calculate OGA activity as nmol/min/µg protein.

### Mitochondrial DNA copy number

Real-Time SYBR Green PCR master mix (Invitrogen) was used to measure the mitochondrial and nuclear 18S DNA [37]. The following primer sequences were used: mtDNA-F (5′-CCAAGGAATTCCCCTACACA-3′), mtDNA-R (5′- GAAATTGCGAGAATGGTGGT-3′), 18S-F (5′-CGAAAGCATTTGCCAAGAAT-3′), and 18S-R (5′-AGTCGGCATCGTTTATGGTC-3′). The mtDNA copy number was normalized to the 18S copy number.

### Mitochondrial DNA damage

Mitochondrial DNA (mtDNA) damage was evaluated by a modified quantitative PCR (QPCR) method [37, 38]. For mtDNA long segment (16 kb), mtLongF (5′-GGA CAA ATA TCA TTC TGA GGA GCT-3′) and mtLongR (5′-GGA TTA GTC AGC CGT AGT TTA CGT-3′) were used. For mtDNA short segment (80 bp), mtShortF (5′-CCAAGGAATTCCCCTACACA-3’) and mtShortR (5′-GAAATTGCGAGAATGGTGGT-3′) were used. The mtDNA long segment and the short segment were amplified using AccuPrime™ Taq DNA Polymerase High Fidelity kit (Life Tech Corp) and separated by agarose gel electrophoresis, respectively. The gels were stained by ethidium bromide, and densitometry analysis was performed using Image J software. Lesion frequency per 16 kb of mtDNA was calculated by the following equation.

$$Lesion frequency per 16 kb= -\mathrm{ln}(\frac{\frac{Long mtDNA}{Short mtDNA}}{Average of \left(\frac{Long mtDNA}{Short mtDNA} from control group\right)})$$

### Western blot analyses

Western blots were performed using antibodies against: O-GlcNAc (Millipore MABA1254, CTD110.6,1:10,000), OGT (Cell Signaling 24083, 1:2000), OGA (Proteintech 14711-1-AP, 1:1000), Citrate synthase (Abcam Ab129095 1:10,000), SOD2 (Abcam ab86087, 1:5000), LDHA (Cell Signaling 2012, 1:5000), HK1 (Abcam ab55144 1:5000), aconitase (produced by Dr. Scott Ballinger, 1:5000), VDAC (Abcam ab61273, 1:5000), glutaminase (Abcam ab156876 1:5000), p62 (Abnova, H00008878-M01,1:5000), Microtubule-associated protein 1 light chain 3 alpha/LC3 (Sigma, L8918, 1:2000), LAMP1 (Abcam 1D4B ab25245, 1:5000), α-synuclein (Santa Cruz SC-7011-R, 1:5000), TPPP (Santa Cruz sc82065, 1:5000), PICALM (Sigma HPA019061, 1:5000), PSD95 (Cell Signaling 3450S 1:5000), synaptophysin (cell signaling 5461, 1:5000), β-actin (Sigma, A5441, 1:5000), GAPDH (Millipore, MAB374,1:5000, or Cell Signaling 36835 1:5000).

### Immunohistochemistry

Half of the brains 3 h after saline or TG administration (n = 3 each male) were placed in 4% paraformaldehyde overnight at 4 °C followed by sucrose, and frozen in OCT blocks and stored at − 80 °C until sectioning. 40 μm sections were used for immunofluorescent staining. The following antibodies were used: O-GlcNAc (UAB core CTD110.6, 1:1000), secondary antibody Alexa fluor 488 (Invitrogen: A-10680, 1:400); OGT (Cell Signaling 24083S, 1:2000), secondary antibody Cy3 (Invitrogen A10520, 1:1000); OGA (Proteintech 14711-1-AP, 1:2000), secondary antibody Cy3 (Invitrogen A10520, 1:1000). Sections were mounted on Fischer SuperFrost Plus microscope slides partially submerged in TBS using a paintbrush. Hoechst nuclear staining was performed. Keyence microscope was used for imaging, and Image J was used for semi-quantitative analyses and Student t-test between saline and TG groups.

### Statistical analysis and visualization

The distribution of dependent variables was assessed using the D’Agostino-Pearson Omnibus normality test of residuals. Between-group comparisons were made using two-way ANOVA to assess differences due to sex, treatment condition, or the interaction of sex and treatment condition. For western blots and mtDNA copy number and damage assays, the Tukey post-hoc multiple pair-wise test was performed. For the OGA activity assay, if a significant difference was identified, multiple comparisons were performed using two-tailed unpaired Student’s t-tests and the false discovery rate (FDR) was controlled using the original FDR method of Benjamini-Hochberg (q* = 0.05). For all statistical analyses, α was set at 0.05. JMP Pro 16 was used for non-parametric Kendall’s rank correlation test. Correlations with Prob >|τ|< 0.05 were considered significant. Cytoscape 3.8.2 was used for visualization of networks of significant relationships with default layout setting (Prefuse force-directed layout).

## Results

### Systemic administration of Thiamet G (TG) inhibits OGA activity and elevates protein O-GlcNAcylation in the brain

**Fig. 1:**
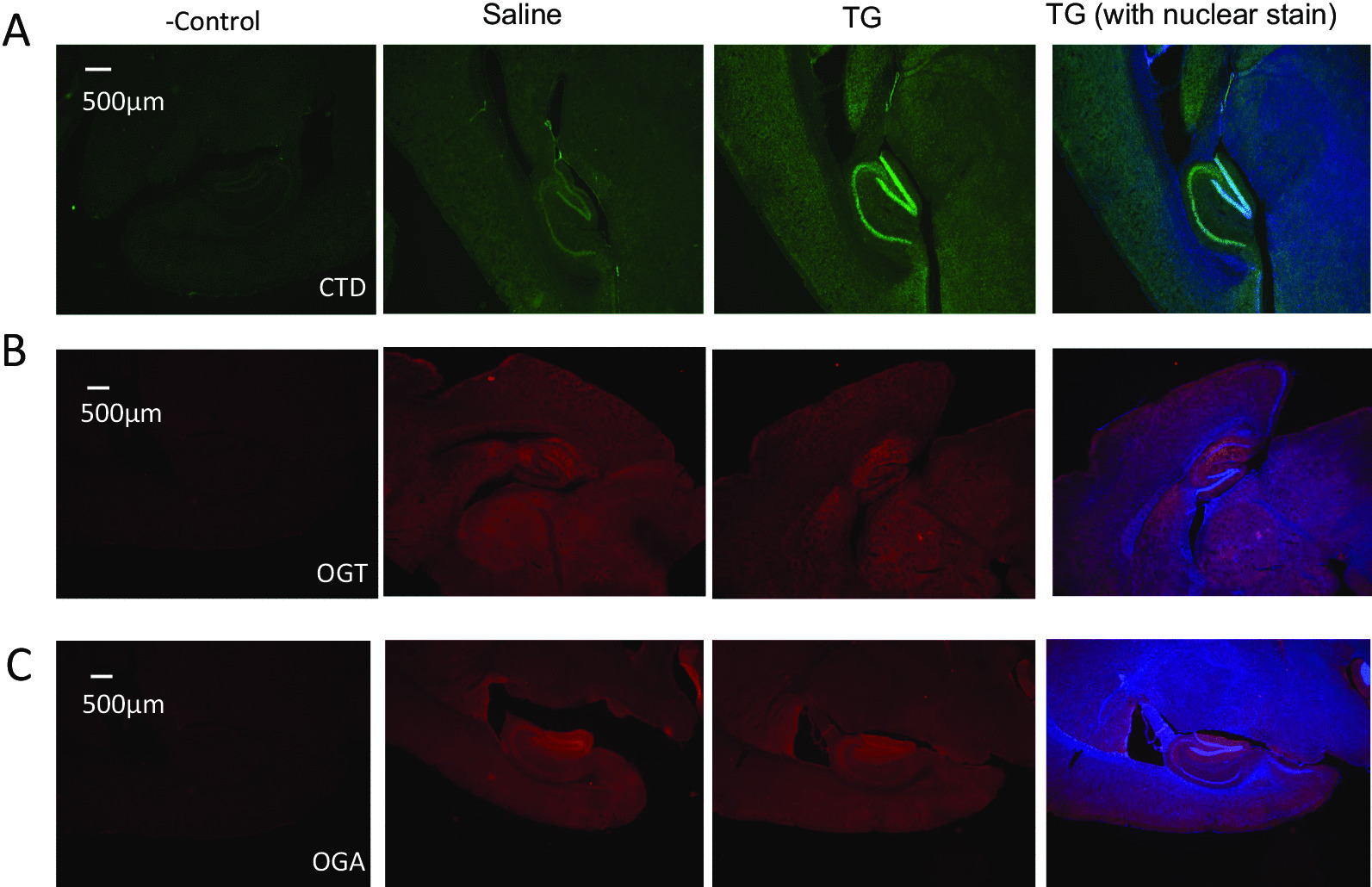
Overall protein O-GlcNAcylation is increased in mouse brains exposed to Thiamet G (TG). **A** Thiamet G increased protein O-GlcNAcylation in broad brain areas. Saline and TG treated mouse brains were dissected and stained with the CTD110.6 anti-O-GlcNAc antibody. **B** Thiamet G did not change OGT immunostaining. Saline and TG treated mouse brains were dissected and stained with an anti-OGT antibody. **C** Thiamet G did not change OGA immunostaining. Saline and TG treated mouse brains were dissected and stained with an anti-OGA antibody. No primary antibody slides were used as negative controls. Representative images from each group were shown. Images were shown without nuclear staining except in the TG group (**A-C**), (n = 3 each). Scale bar = 500 µm

Male mice 2 months of age were administered saline or TG (10 mg/kg i.p.) and brain samples were harvested 3 h later. Half of the brains were processed for immunohistochemistry with O-GlcNAc, OGT and OGA antibodies (n = 3 each group) (Fig. 1). Significant increases of O-GlcNAcylated proteins were found in the TG group both in the cortex and in the hippocampus (both total and CA1 immunoreactivity were significantly increased to ~ 1.8 and 1.9 fold, respectively, p < 0.05), with intense nuclear staining (Fig. 1A, Additional file 1: Figs. S1, S2), consistent with the observations that nuclear proteins are heavily O-GlcNAcylated. There were no significant changes in OGT and OGA protein levels (Fig. 1B, C, Additional file 1: Figs. S3–S6). Compared to immunostaining of O-GlcNAcylated proteins by CTD110.6 antibody, OGT immunostaining appears to be more cytosolic with less intense immunoreactivity in hippocampal CA1 (ratios of immunoreactivity intensity between CA1 to adjacent region are ~ 0.73 for OGT and ~ 2.5 for CTD110.6, respectively) or dentate gyrus neurons compared to the areas with less dense nuclei (Fig. 1B, Additional file 1: Figs. S3, S4). OGA staining appears to be between CTD110.6 and OGT immunostaining that the ratio of OGA immunoreactivity intensity between CA1 to adjacent regions is ~ 1.3 and that OGA immunostaining is partially overlapping with dentate gyrus nucleus (Fig. 1C, Additional file 1: Figs. S5, S6).

**Fig. 2:**
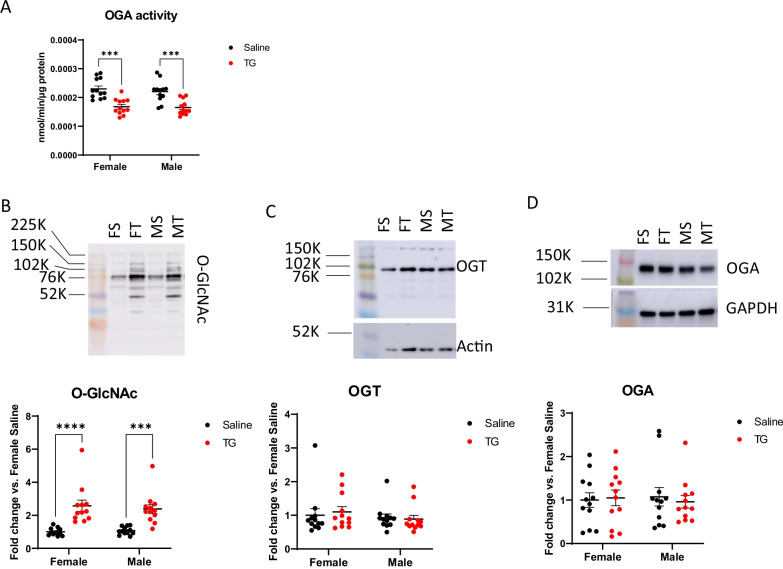
OGA activity is decreased in mice exposed to Thiamet G (TG). **A** OGA activity assay in cortical homogenates from male and female mice treated with either vehicle (0.9% saline; Sal) or TG. The rate of 4MU release over time was analyzed by two-way ANOVA and sex differences assessed by Student’s t-tests and false discovery rate controlled using the Benjamini–Hochberg method (q* = 0.05). ***p < 0.0001. **B** Representative image and western blot analysis of O-GlcNAcylated protein levels in saline-treated females (FS), TG-treated females (FT), saline-treated males (MS), and TG-treated males (MT) measured with the CTD110.6 antibody. ***p < 0.001, and ****p < 0.0001 compared to saline controls by student t-test (2-way ANOVA and post-hoc Tukey). **C, D** Representative image and western blot analyses of OGT and OGA protein levels

Next, we performed a study with both sexes and n = 12 mice each sex each treatment group (saline versus TG) (10 mg/kg i.p.) and brain samples were harvested 3 h later. Cortical protein extracts were prepared and used to assess OGA activity and revealed a significant decrease of approximately 25% which was similar between males and females (Fig. 2A). The overall protein O-GlcNAcylation was determined by western blot analyses using the CTD110.6 antibody. We found that there was a significant increase in the overall protein O-GlcNAcylation in both males and females (Fig. 2B). Using the same protein lysates, TG administration did not significantly change the OGT and OGA protein levels (Fig. 2C, D).

### Kendall’s correlation analysis of O-GlcNAcylation related proteins with metabolic/autophagy/neurodegenerative disease-related markers in males and females with and without TG. 

We performed mitochondrial electron transport activity measurements of all cortical samples using the Seahorse XF analyzer (Additional file 1: Fig. S7). In addition, we measured citrate synthase and lactate dehydrogenase activities using biochemical methods (Additional file 1: Fig. S8). Proteins related to metabolism and mitochondrial function were examined by western blot analyses (Additional file 1: Fig. S9). Mitochondrial DNA (mtDNA) copy number and damage were analyzed by quantitative PCR (Additional file 1: Fig. S10). Autophagy-related proteins and proteins implicated in neurodegenerative diseases were also examined (Additional file 1: Figs. S11, S12). There are overall 50% to 400% changes (coefficient of variations CV% within each group) between the highest measured values and the lowest measured values in the measurements within the same group, with citrate synthase protein levels and activities being the least variable, and no between-group differences (Additional file 1: Figs. S7–S12). We found that each animal has its unique value for OGT, OGA, OGA activity, mitochondrial activities and parameters of autophagy proteins and proteins involved in neurodegenerative diseases. Even with the same sex and under control conditions, some of the parameters exhibit a twofold difference in OGA, CS or mitochondrial ETC activities, as well as autophagy and neurological disease-related protein levels.

We hypothesized that even though some of the single parameters were not significantly different between groups, bi-variant relationships between autophagy, bioenergetic, neurodegenerative disease-related, or O-GlcNAcylation parameters could be significant in either the female or the male group, however not both groups.. For example, this can occur if the regulation of the O-GlcNAcylation pathway is sex dependent as has been suggested [39, 40]. Furthermore, since one of the functions of O-GlcNAcylation is proposed to integrate the activities of multiple signaling pathways, bi-variant analyses may help identify pathway alterations related to altered O-GlcNAcylation that may not be evident when assessing dependent measures independently. To test this we used the Kendall’s correlation analysis on all parameters within each group, in either sexes, with and without TG treatment (Additional file 1: Tables S1–S6, Additional file 2: Fig. S13). Significant relationships (Prob >|τ|< 0.05) were then used for the subsequent analysis and discussion.

**Fig. 3:**
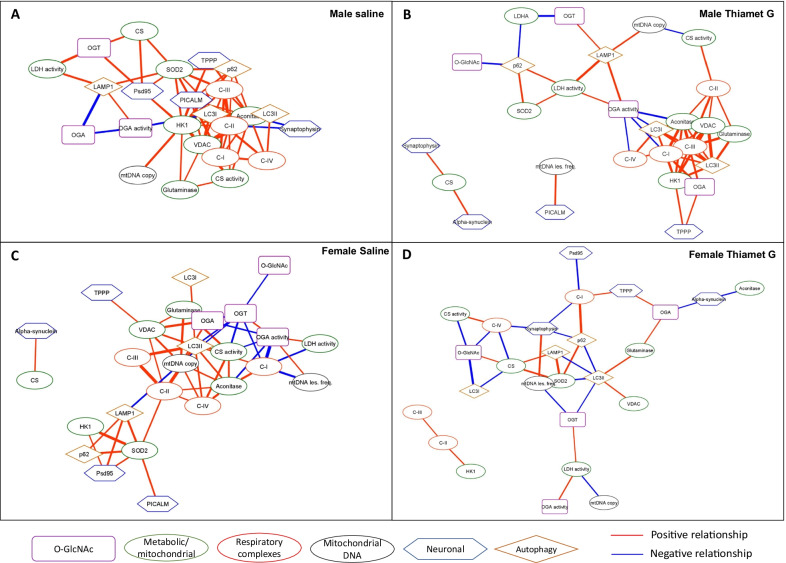
Correlation networks for **A** Male saline, **B** Male Thiamet G, **C** Female Saline, **D** Female Thiamet G. Kendall’s rank correlation tests were performed. All correlations are significant with Prob >|τ|< 0.05. Network visualization was performed using Cytoscape 3.8.2. The nodes are grouped based on the functions of the proteins or the localizations or the processes involved. Red edges denote positive relationships, whereas blue edges denote negative relationships. The thickness of the edges represents the strength of the relationships based on the Kendall Tau coefficient magnitude (Additional file 3: Tables S2–S5)

Shown in Fig. 3 are the networks for all significant relationships for each experimental group, with positive relationship (when one is higher the other is also higher) and negative relationship (when one is higher the other is lower) depicted in red and blue respectively. The thickness of the lines directly correlates with the Kendall Tau coefficient magnitude, indicating the strength of the relationship. In the male saline group, there are 62 total significant relationships with 57 being positive and 5 negative centered on metabolic nodes (hexokinase 1 (HK1), complexes I, II, III, and IV, superoxide dismutase 2 (SOD2), aconitase, citrate synthase (CS) activity, voltage-dependent anion channel (VDAC), as well as phosphatidylinositol binding clathrin assembly protein (PICALM), an Alzheimer’s disease risk protein involved in autophagy and endocytosis. In males following TG treatment, the number of total relationships decreases to 56, with 48 being positive and 8 negative, although the strength of most of the relationships did not change (Fig. 3A, B, Additional file 3: Table S2, S3). In the female saline group, there are 51 significant relationships, with 38 being positive and 13 negative; with OGA activity, microtubule-associated protein 1A/1B-light chain 3-II (LC3II), VDAC, mtDNA copy, mitochondrial electron transport chain complexes I, II, III, and IV activities, VDAC, and aconitase as prominent nodes. After TG treatment, the total number of relationships decreases to 34 (28 being positive and 13 negative), with synaptophysin and LC3II as prominent nodes, many lines were thinner indicating weaker relationships with a lower Tau coefficient magnitude (Fig. 3C, D, Additional file 3: Table S4, S5).

### Association of metabolic/autophagy/neurodegenerative disease-related markers with O-GlcNAcylation in males and females with and without TG. 

**Fig. 4:**
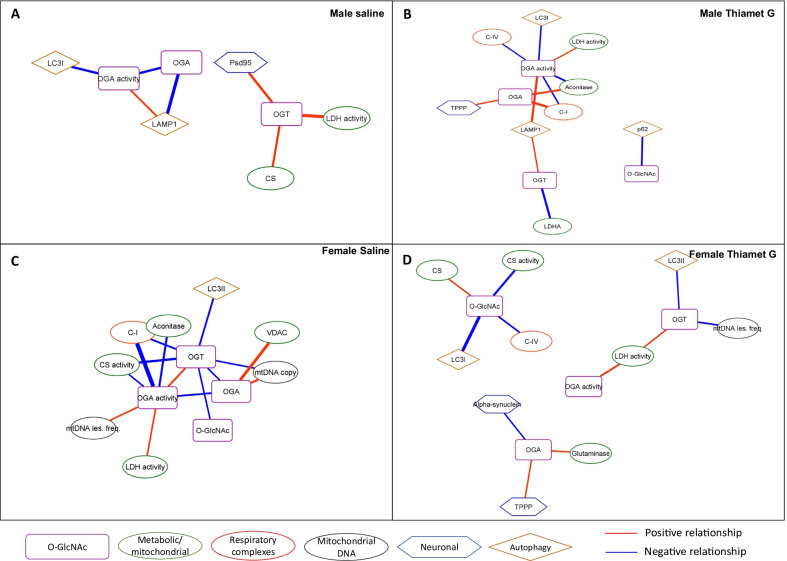
Networks for variables with significant correlations with O-GlcNAc related variables. **A** Male saline, **B** Male Thiamet G, **C** Female Saline, **D** Female Thiamet G. Kendall’s rank correlation tests were performed. All correlations are significant with Prob >|τ|< 0.05. Network visualization was performed using Cytoscape 3.8.2. The nodes are grouped based on the functions of the proteins or the localizations or the processes involved. Red edges denote positive relationships, whereas blue edges denote negative relationships. The thickness of the edges represents the strength of the relationships based on the Kendall Tau coefficient magnitude (Additional file 3: Tables S2–S5)

To examine the interrelationships in more depth, we show in Fig. 4 the panel of O-GlcNAcylation-related parameters (protein O-GlcNAcylation levels, OGA and OGT levels, and OGA activity) selected from Fig. 3 and their connection networks. In the male saline group, there are relatively few edges linking O-GlcNAc related parameters (Fig. 4A). OGA activity shows a significant negative relationship with OGA protein levels and autophagy-related protein LC3I, and a positive relationship to LAMP1. These relationships are further illustrated in Additional file 2: Figs. S14, S15. OGT is positively correlated to citrate synthase protein levels, lactate dehydrogenase (LDH) activity and postsynaptic density protein 95 (PSD95), which is an important postsynaptic scaffolding protein and decreased levels are associated with Alzheimer disease pathology [41–43].

Since treatment with TG led to partial inhibition of OGA (Fig. 2A), we hypothesized that the decreased activity of OGA would result in pathways regulated by O-GlcNAcylation to become more sensitive to OGA activity. Consistent with this, the number of correlations with OGA activity increased from 3 to 6 in the male TG treatment group compared to the control (Fig. 4B). The autophagy markers LC3I and LAMP1 maintained their relationships with OGA activity. A new relationship emerged between OGA levels and tubulin polymerization-promoting protein (TPPP), a protein involved in tubulin stabilization and has been linked to neurodegenerative diseases [44–46]. A new relationship also emerged between sequestosome-1 or ubiquitin-binding protein p62 and protein O-GlcNAcylation levels. This correlation suggests that higher O-GlcNAcylation may result in p62 degradation consistent with increased autophagic flux, while it may also be possible that p62 production is suppressed consistent with a decrease in autophagic flux. Most striking was the increase in the numbers of metabolic proteins which emerged as now being negatively correlated to OGA activity including the activities of mitochondrial complexes I, and IV; and aconitase levels; and positively correlated with LDH activity. In addition to OGA activity, OGT protein levels also show a positive association with LAMP1.

Similarly, the panel of O-GlcNAcylation-related parameters (protein O-GlcNAcylation levels, OGA and OGT levels, and OGA activity) selected from Fig. 3 and their connection networks were analyzed in females (Fig. 4C, D). Strikingly, instead of having a connecting cluster as seen in the males with OGA activity/OGA protein levels connected to LC3I and LAMP1, and another independent connecting cluster with OGT protein levels connected to LDH activity, citrate synthase and PSD95 levels; in female, OGA activity/OGA protein level/OGT protein levels form a single cluster. Instead of OGA activity, OGT protein level is negatively correlated with LC3II, suggesting the different regulatory relationship in the autophagy/lysosomal pathway between the two sexes. In contrast to the male saline group, OGA activity is a prominent node with 7 edges compared to only 3 in the control males (Fig. 4C). As in the male saline, OGA protein levels are negatively correlated with OGA activities. But in the female saline group, OGT protein levels are positively correlated with OGA activities. Protein O-GlcNAcylation levels are negatively correlated with OGT levels, and OGT levels are negatively correlated with OGA levels. The negative correlations of OGA activity with proteins related to metabolic pathways, which only emerge with TG treatment in the males, are evident in the female saline group, with mitochondrial complex I activities, aconitase level, citrate synthase activity, and mtDNA lesion. OGT protein levels show a negative relationship with complex I and citrate synthase activities, and mtDNA copy number. The outer mitochondrial membrane protein VDAC levels are positively correlated with the levels of OGA protein, which are positively associated with mtDNA copy number. The associations of mtDNA copy number, mtDNA lesion, and mitochondrial enzyme activities with OGA activity and OGT protein levels further support previous findings suggesting an important role for the O-GlcNAc pathway in regulating autophagy [16, 24].

Treatment of the female group with TG markedly decreased the correlations of metabolic proteins related to OGA activity; with the only significant association being with LDH activity. OGA levels lost all previous edges, replaced by 3 new edges with TPPP, α-synuclein, and glutaminase levels (Fig. 4C, D). Similarly, OGT exhibits only 3 edges which now include a negative correlation with mtDNA damage. The level of protein O-GlcNAcylation measured by western blot in TG-treated females shows a positive correlation with citrate synthase protein levels, and a negative correlation with citrate synthase and complex IV activities. There is a strong negative association of O-GlcNAc protein levels with LC3-I (Additional file 1: Fig. S16). Taken together, these data are consistent with high levels of O-GlcNAcylation correlating with less functional mitochondria in the presence of higher citrate synthase protein levels in female mice following TG treatment.

### Association of O-GlcNAcylation/autophagy/neurodegenerative disease-related markers in males and females with and without TG with metabolic parameters 

**Fig. 5:**
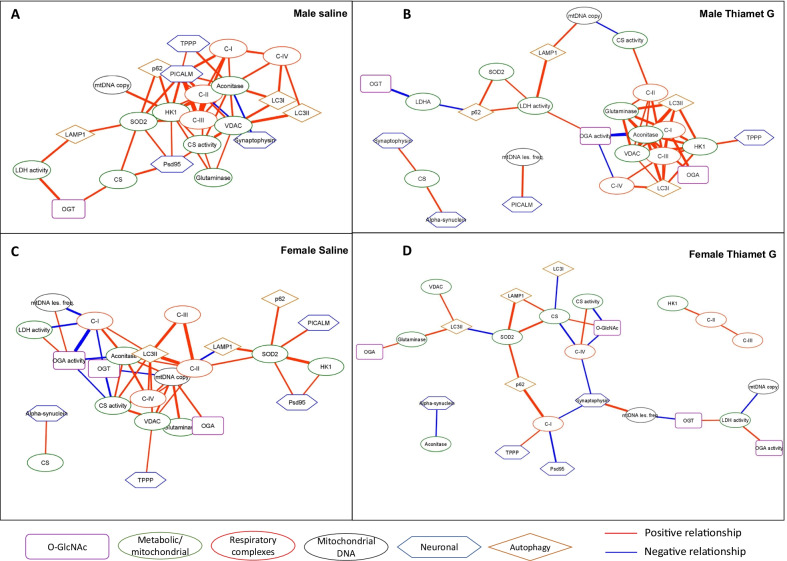
Networks for variables with significant correlations with variables related to metabolic/mitochondrial processes and respiratory complexes. **A** Male saline, **B** Male Thiamet G, **C** Female Saline, **D** Female Thiamet G. Kendall’s rank correlation tests were performed. Kendall’s rank correlation tests were performed. All correlations are significant with Prob >|τ|< 0.05. Network visualization was performed using Cytoscape 3.8.2. The nodes are grouped based on the functions of the proteins or the localizations or the processes involved. Red edges denote positive relationships, whereas blue edges denote negative relationships. The thickness of the edges represents the strength of the relationships based on the Kendall Tau coefficient magnitude (Additional file 3: Tables S2–S5)

In this analysis, we highlight the sub-network data from Fig. 3 related to metabolic/mitochondrial parameters. In the male saline group (Fig. 5A), the metabolic enzymes are tightly clustered with strong positive correlations among the activity of complexes I-IV, mtDNA copy number, matrix enzymes such as citrate synthase level and activity, as well as VDAC, which serves multiple functions including substrate transport and control of apoptosis [47–49]. Taken together these data are consistent with concurrent control of mitochondrial mass and function. The connection between autophagy and mitochondrial mass and activity is supported by the positive correlation of LC3-I and LC3-II with complex IV and VDAC. Notably, the glycolytic enzyme HK1 has a central position in this network consistent with its association with neuronal mitochondria [50, 51]. Complex IV is correlated with complex I activity and the protein level of the mitochondrial TCA cycle enzyme, aconitase. Complex III is correlated with the protein levels of SOD2, which is consistent with the important role this enzyme plays in the generation of mitochondrial hydrogen peroxide. In addition to the expected relationships between SOD2 and mitochondrial complexes III activities, there are also positive correlations between SOD2 with protein levels of CS, PSD95, PICALM, and p62. PSD95 is an important postsynaptic scaffolding protein and decreased levels are associated with Alzheimer’s disease pathology [41–43]; PICALM is involved with endocytosis and Alzheimer’s disease risk [52–55]; while p62 plays a key role in autophagy [31].

In the male TG group, the relationships among metabolic proteins and activities form a cluster with OGA activity as a central node (Fig. 5B). The positive correlations between mitochondrial metabolic enzymes and LC3-I and LC3-II are maintained, while OGA activity is negatively correlated to aconitase protein level and complex I and IV activities; and positively correlated to LDH activities. In addition, LAMP1 exhibits a positive correlation with mtDNA copy number. Citrate synthase levels are positively correlated with α-synuclein and synaptophysin levels, and mtDNA damage is positively correlated with PICALM levels.

Similar to the male saline group, the female saline group also exhibits relationships between mitochondrial electron transport chain complex activities, with complexes I, II, III, and IV in a cluster associated with aconitase, LC3II, CS activity, and VDAC (Fig. 5C, Additional file 2: Fig. S17). Unlike the male saline group, HK1 is only associated with SOD2 and psd95. In addition, OGA activities gained positive associations to mtDNA lesion and LDH activity, and negative associations to aconitase level, and Complex I and CS activities. SOD2 is a prominent node in both male and female saline groups; positively associated with levels of HK1 [50, 51], autophagy proteins p62 and LAMP1 [31], and neurodegenerative disease-associated proteins, PSD95 [41–43] and PICALM [52–55], except that in the female saline group, SOD2 is correlated with complex II but not complex III activity.

In contrast to the male group, where the effects of TG treatment appear to enable positive relationship between LDH activity and OGA activity, and negative relationships between complexes I and IV activities, and OGA activity; in females, there is a weakening of the networks between metabolic enzymes or autophagy with O-GlcNAc parameters, with Complexes I and IV being in one cluster, and Complexes II and III being in another independent cluster (Figs. 3D, 5D). Similar to the males, after TG treatment, SOD2 still has a positive association with p62 after TG treatment; while in females, SOD2 exhibits positive relationships with CS and LAMP1 levels, and a negative relationship with LC3II levels. In addition, TG treatment in the female enabled a negative relationship between aconitase and α-synuclein levels, which was a positive association in the female saline group.

### Association of O-GlcNAcylation/metabolic parameters with autophagy/neurodegenerative disease-related markers in males and females with and without TG

**Fig. 6:**
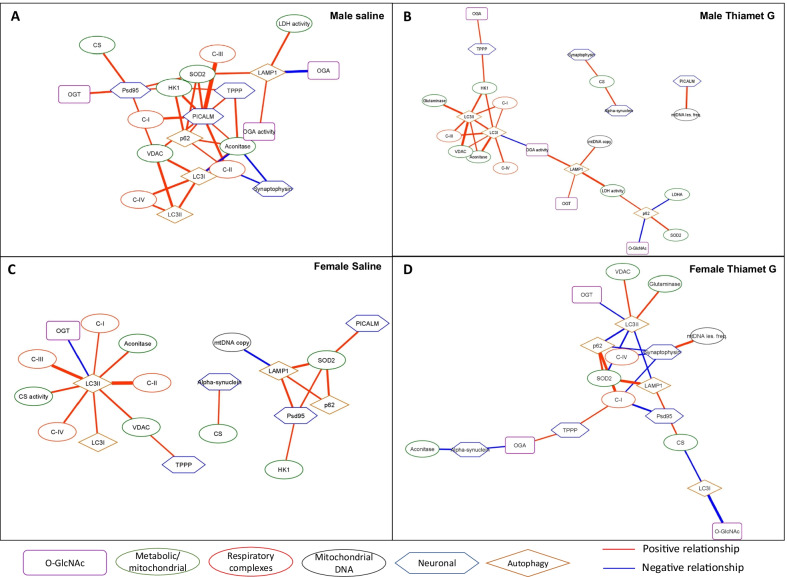
Networks for variables with significant correlations with variables related to neuronal or autophagy processes. **A** Male saline, **B** Male Thiamet G, **C** Female Saline, **D** Female Thiamet G. Kendall’s rank correlation tests were performed. All correlations are significant with Prob >|τ|< 0.05. Network visualization was performed using Cytoscape 3.8.2. The nodes are grouped based on the functions of the proteins or the localizations or the processes involved. Red edges denote positive relationships, whereas blue edges denote negative relationships. The thickness of the edges represents the strength of the relationships based on the Kendall Tau coefficient magnitude (Additional file 3: Tables S2–S5)

Finally, we highlight from Fig. 3 the networks related to autophagy and the neuronal markers TPPP, synaptophysin, PSD95, PICALM, and α-synuclein (Fig. 6). As part of the autophagy machinery, LAMP1 is positively associated with SOD2 in saline groups of both sexes, while such connection remained in female TG group, it is replaced with mtDNA copy in the male TG group. In both male saline and TG groups, LAMP1 is associated with LDH activity, while such relationship is not significant in the female groups. The level of p62 correlates with Complex II activity, levels of aconitase, VDAC, HK1, PICALM, and SOD2 in male saline group, while only with LAMP1 and SOD2 in female saline group. After TG treatment, p62 in the male is positively correlated to SOD2 level and LDH activity, and negatively with LDH protein level and overall protein O-GlcNAcylation. In contrast, in the female TG group, p62 is positively associated with SOD2 level and complex I activity, and negatively associated to LC3-II and synaptophysin levels.

In the male saline group, PICALM forms a cluster with strong positive associations with complexes I-III activities, protein levels of HK1, and SOD2 (Fig. 6A, Additional file 2: Fig. S18). PICALM is also positively associated with p62 and TPPP, proteins involved in autophagy, and has previously been shown to be involved in the clearance of β-amyloid as well as iron transport essential for mitochondrial function [52–56]. PSD95 forms a cluster with positive relationships with CS, OGT, SOD2, and HK1 levels, and complex I activity, suggesting an interaction with mitochondrial function and ROS regulation. Synaptophysin is negatively correlated with complex II activity and aconitase. Interestingly, synaptophysin is a synapse-associated protein proposed to contribute to behavioral changes including spatial learning and protein O-GlcNAcylation has been shown to affect synaptophysin levels and modify both behavioral and synaptic function [57]. Treatment with TG in males disrupted the network by diminishing the PICALM relationship network, which only has one association with mtDNA lesion frequency. In addition, LC3-I and LC3-II form a prominent cluster, that is positively associated with complexes I, III and IV activities, aconitase, VDAC and HK1 protein levels and negatively associated with OGA activities, consistent with a strong relationship between autophagy and mitochondrial function (Fig. 6B).

The female saline group exhibits a prominent cluster with LC3-II positively associated with LC3-I levels and metabolic enzymes (complex I-IV activities, CS activity, aconitase, and VDAC levels), and negatively associated with OGT protein levels. The PICALM network evident in the male saline group is much less prominent in the female saline group, with only one positive relationship with SOD2. The CS level relationship with α-synuclein level noted in the TG-treated males is also evident in the female saline group. Treatment with TG in females disrupted the LC3-II network with only positive association with VDAC and negative association with OGT remaining. In addition, LC3II positive relationships with glutaminase and negative relationships with SOD2, LAMP1, and p62 emerged upon treatment with TG. Synaptophysin is negatively correlated with complexes I and IV activities, p62 levels, and positively correlated with mtDNA lesion frequency, linking accumulation of mitochondrial damage to an accumulation of synaptophysin. α-synuclein is negatively correlated with aconitase and OGA protein level.

## Discussion

The modification of proteins by O-GlcNAc not only contributes to the regulation of diverse cellular functions, such as mitochondrial function, autophagy, and cell survival, but is also associated with a number of neurodegenerative diseases including Alzheimer’s and Parkinson’s diseases [2, 16, 17]. As a result, there has been considerable interest in identifying changes in the O-GlcNAc levels of specific proteins in models of such diseases as well as the development of therapeutics to change overall O-GlcNAc levels. However, O-GlcNAcylation is a dynamic process and several studies have shown that acute increases in O-GlcNAcylation through inhibition of OGA can rapidly induce changes in neurological function [16, 25–27]. Moreover, we have recently identified 65 proteins that exhibited a significant increase in O-GlcNAc levels after only 3 h following a single treatment with the OGA inhibitor thiamet G [35]. It is important to understand the physiological consequences of these acute changes in O-GlcNAcylation in the brain in order to develop a better understanding of the implications of dysregulation in O-GlcNAc cycling in chronic neurodegenerative diseases. Therefore, we examined the impact of 3 h of OGA inhibition in male and female mouse brains on metabolic, bioenergetic, mitochondrial, autophagic, neurodegeneration-related, and O-GlcNAc-related parameters. To identify relationships and potential networks we used Kendall’s bivariate method in an independent analysis within each group to reveal correlations between autophagy, bioenergetic and O-GlcNAcylation parameters in the male and female groups with and without TG. We identified novel pathways linking metabolism, mitochondria and autophagy that were changed in an O-GlcNAc dependent manner. Our findings also revealed for the first time a strong sex dependent effect on the interactions between O-GlcNAcylation, mitochondrial function, and mitochondrial quality control. These results demonstrate the importance of understanding the inter-relationship of O-GlcNAc-dependent pathways in the brain as a complement to more traditional studies of the effects of O-GlcNAcylation on specific proteins and their function.

Our focus on short-term pathway interactions following OGA inhibition captures the acute physiological consequences of increased O-GlcNAcylation, limiting the effects of O-GlcNAc on changes in transcription, translation, and protein degradation that can occur following longer treatment periods. This acute treatment paradigm allows examination of rapid cellular alterations which take place prior to the establishment or maintenance of a new level of O-GlcNAc homeostasis that occur in chronic or prolonged thiamet G treatment conditions. Within 3 h following treatment with TG there was an approximately 25% decrease in OGA activity in the cortex in both male and female groups. Using both immunostaining and western blotting to assess total levels of O-GlcNAcylated protein we found that there was a significant increase in overall protein O-GlcNAc levels, consistent with OGA inhibition as previously reported using TG (Figs. 1, 2). There was a lack of a direct relationship between total O-GlcNAc levels and OGA activity across all groups, which is consistent with the fact that both OGA and OGT activities regulate protein O-GlcNAc levels.

We recently performed a proteomics study in male mice with and without thiamet G using the same treatment paradigm. In the proteomics study, we have not found any of the mitochondrial proteins with altered O-GlcNAcylation 3 h following thiamet G treatment [35]. However, we found that Alzheimer’s disease risk factor PICALM, is O-GlcNAcylated and its O-GlcNAcylation is upregulated by thiamet G [35]. OGT, TPPP and α-synuclein are also among those detected to be O-GlcNAcylated, although we did not see any changes of their O-GlcNAcylation levels in response to thiamet G. In addition, the O-GlcNAcAtlas (https://oglcnac.org) reports that OGA, OGT, LAMP1, TPPP, PICALM, α-synuclein, HK1, VDAC, aconitase, glutaminase, and various subunits of the mitochondrial electron transport chain complexes I, II, III, and IV identified in our network analyses are also O-GlcNAc targets in either human or mouse cells/tissues (Additional file 3: Table S6). In a curated database of OGT proteins interacting network (OGT-PIN) based on the 929 proteins from human, mouse, rat and flies [58], OGA, p62, PICALM, LDHA, HK1, VDAC, glutaminase, subunits of the mitochondrial electron transport chain complexes I, III, and IV, were also reported to interact with OGT [58]. A database for OGA interacting proteins does not currently exist; however, Groves et al. identified 90 OGA interacting proteins using BioID proximity biotinylation in combination with stable isotopic labeling of amino acids in cell culture (SILAC), under basal and stress conditions [59]. Except for OGT which was found to interact with OGA by co-IP/western blot analyses, none of the proteins we examined in the networks corresponded to those identified in Groves’ study [59], most likely due to the limited number of OGA interacting proteins reported.

In each of the network analyses we observed a surprisingly marked difference between sexes under control saline conditions as well as in their responses to TG treatment. Interestingly, only in the female saline group were OGT protein levels negatively associated with OGA protein levels and overall protein O-GlcNAcylation, and positively associated with OGA activities. It is possible that these observed sex differences are a consequence of the fact that the *Ogt* gene is located on the X chromosome; however, further studies are needed to examine this in more detail [60]. Our observed sex differences in the correlation networks and the response to TG may be partially related to the sex-dependent glycomes, which is supported by differential O-GlcNAc glycoproteomics between male and female flies in response to OGA mutation, although none of the proteins in our study was detected in the fly glycoproteomics (oga^del.1^) [61], likely due to differences in species, tissue and age. Sex-dependent differences in brain metabolism have been previously reported; for example, NAD^+^ metabolism is different between the two sexes in 3xTg Alzheimer’s disease mouse brain [62]. This is interesting as both glycolysis and mitochondria are critical for maintaining the NAD/NADH redox couple. In our study, in males, LDH activity, which controls NAD^+^ availability for glycolysis, was positively correlated with LAMP1 levels in both saline and TG, suggesting an association between glycolytic activity and lysosomal mass. In females LDH activity was correlated with OGA activity in both saline and TG, also suggesting a coordinated activity between the glycolytic and the O-GlcNAc pathway. Additionally, in male saline treated mice LDH activity was linked with OGT; however, this relationship was disrupted following TG treatment and instead LDH activity was associated with SOD2, p62, and OGA activity. On the other hand, in female saline mice LDH activity was negatively associated with complex I activity which was disrupted following TG; however, after TG treatment there was a positive association of LDH activity with OGT and negative association with mtDNA copy number. These changes suggest that in males inhibition of OGA by TG enables a closer association between LDH activity with autophagy machinery, and mitochondrial antioxidant signaling. In females TG treatment disrupted the relationship of LDH activity with mitochondrial function and established a closer relationship with the O-GlcNAc pathway.

The sex differences seen here were under basal (saline) and acute TG treatment; however, sex differences are also observed in aging, and age-related neurodegenerative diseases. Although our understanding of the effects of sex on these processes is only slowly emerging, gene expression has been shown to be different in male and female human tissues, including multiple brain regions [63]. There was a previous report demonstrating differences between sexes in the autophagy responses to starvation and Atg7 knockdown [64]. As reported in Additional file 3: Table S6 that in earlier studies, LAMP1, TPPP, PICALM, and alpha-synuclein, are O-GlcNAcylated, and that p62 and PICALM, have been shown to interact with OGT [2, 35, 58]. Our finding that significant differences in the relationships among LAMP1, p62, TPPP, PICALM and alpha-synuclein are sex-dependent and altered by TG is consistent with a sex- and O-GlcNAc-dependent regulation of autophagy and neurodegenerative disease pathogenesis and potential response to therapeutics. Our findings suggest that the networks of O-GlcNAc with metabolism, autophagy and neuronal proteins may be another important factor to consider for sex-dependent phenotypes in aging and neurodegenerative diseases.

Interestingly we found that SOD2 has extensive positive correlations with PICALM, p62, LAMP1, complex III, CS and PSD95 in the male saline group, but only with LDH activities and p62 in the male TG group. Whereas in females, SOD2 is positively correlated with PICALM, p62, complex II, PSD95, HK1, and LAMP1 in the saline group, and is also positively correlated with p62, LAMP1, and CS, and negatively with LC3II, following TG treatment. Overall, most of the SOD2 connections appear to be outside of the mitochondria, consistent with the hypothesis that SOD2 provides a link conveying signals from the mitochondria to other parts of the cell via hydrogen peroxide signaling. As mentioned above and as shown in Additional file 3: Table S6, HK1, VDAC, aconitase, glutaminase, and various subunits of the mitochondrial electron transport chain complex I, II, III, and IV, have been reported to be O-GlcNAcylated. Furthermore, LDHA, HK1, VDAC, glutaminase, subunits of the mitochondrial electron transport chain complexes I, III, and IV, have all been shown to interact with OGT [58]. While many components of the mitochondrial electron transport chain have been shown to be O-GlcNAcylated, the consequence of this modification on mitochondrial function remains poorly understood. For example, two studies showed that increased O-GlcNAcylation in cardiac cells or tissues correlated with decreased electron complex activities, while two other studies showed that increased O-GlcNAc attenuated mitochondrial Ca^2+^ uptake [28, 30, 65]. Such controversial observations are likely due to differences in O-GlcNAc modulation approaches, treatment duration, and experimental models.

The positive and negative correlations in the networks of each group, while providing insights into novel relationships between O-GlcNAcylation, bioenergetics and autophagy are not intended to demonstrate causation. Rather these results provide a foundation for future studies in which causal relationships between the various nodes can be investigated. For example, manipulation of LAMP1 levels followed by quantification of mtDNA copy number can validate the relationship between these two parameters. Likewise, it can be of interest to determine the effects of altered citrate synthase levels on α-synuclein and synaptophysin levels, or of different PICALM levels on mtDNA integrity.

In conclusion, in the brain we used an unbiased approach to examine the interconnections between mitochondrial function, autophagy and neurodegenerative disease related proteins and how these connections were altered by sex and acute increases in O-GlcNAc levels via OGA inhibition. The advantage of this analysis is that it can reveal new potential areas for future research. For example, the analysis of the male and female groups revealed sex-dependent nodes responsive to acute increases in O-GlcNAc levels brought about by OGA pharmacological inhibition. While a number of studies have examined the potential role of O-GlcNAcylation in chronic neurological conditions, our goal was to examine short term changes in O-GlcNAc levels in the brain, which we can then provide a foundation for better understanding the long term physiological and pathophysiological consequences. As such we anticipated that the magnitude of changes in individual parameters would likely be small but that correlation analyses within groups would reveal changes in relevant metabolic networks. We identified a number of novel relationships between O-GlcNAcylation, metabolism, mitochondrial function, and autophagy and showed important sex-dependent effects on these interactions. Importantly, many of the specific proteins identified as key network nodes have been shown to either be targets for O-GlcNAcylation or OGT interacting proteins. While these are correlations studies and as such cannot demonstrate direct causation, this unbiased approach provides new insights on how O-GlcNAc cycling interacts with networks that have a potential role in the development of neurodegenerative diseases. We anticipate that the identification of these networks will provide the basis for more mechanistic studies into how O-GlcNAcylation regulates these processes under normal and pathological conditions, and will contribute to the development of therapeutic approaches to modulate O-GlcNAc levels in the brain.

## Supplementary Information

**Additional file 1: Figure S1.** Higher magnification images of CTD antibody immunostaining demonstrating that overall protein O-GlcNAcylation is increased in the cortex exposed to Thiamet G (TG). No primary antibody was used as a negative control. Representative images mouse from saline and TG group were shown (n = 3 each). Scale bar = 100 µm. Blue: nuclear staining. Green: CTD staining. Merge: both nuclear and CTD staining. **Figure S2.** Higher magnification images of CTD antibody immunostaining demonstrating that overall protein O-GlcNAcylation is increased in the hippocampus exposed to Thiamet G (TG). No primary antibody was used as a negative control. Representative images mouse from saline and TG group were shown (n = 3 each). Scale bar = 100 µm. Blue: nuclear staining. Green: CTD staining. Merge: both nuclear and CTD staining. **Figure S3.** Higher magnification images of anti-OGT antibody immunostaining in the cortex. No primary antibody was used as a negative control. Representative images mouse from saline and Thiamet G (TG) group were shown (n = 3 each). Scale bar = 100 µm. Blue: nuclear staining. Red: OGT staining. Merge: both nuclear and OGT staining. **Figure S4.** Higher magnification images of anti-OGT antibody immunostaining in the hippocampus. No primary antibody was used as a negative control. Representative images mouse from saline and Thiamet G (TG) group were shown (n = 3 each). Scale bar = 100 µm. Blue: nuclear staining. Red: OGT staining. Merge: both nuclear and OGT staining. **Figure S5. **Higher magnification images of anti-OGA antibody immunostaining in the cortex. No primary antibody was used as a negative control. Representative images mouse from saline and Thiamet G (TG) group were shown (n = 3 each). Scale bar = 100 µm. Blue: nuclear staining. Red: OGA staining. Merge: both nuclear and OGA staining. **Figure S6.** Higher magnification images of anti-OGA antibody immunostaining in the hippocampus. No primary antibody was used as a negative control. Representative images mouse from saline and Thiamet G (TG) group were shown (n = 3 each). Scale bar = 100 µm. Blue: nuclear staining. Red: OGA staining. Merge: both nuclear and OGA staining. **Figure S7.** Mitochondrial complexes I-IV substrates linked activities in cortical samples from mice administered saline or thiamet G. We measured mitochondrial respiration linked to the complex I-IV (A-D) substrates, using the Seahorse XF analyzer. The same amount of proteins were used for the measurements. Oxygen consumption rate (OCR; pmol/min/4 µg protein) is shown for each mouse. FS: female saline. FT: female thiamet G. MS: male saline. MT: male thiamet G. **Figure S8.** Citrate synthase and lactate dehydrogenase (LDH) activities. We measured (A) mitochondrial matrix enzyme citrate synthase, and (B) cytosolic enzyme lactate dehydrogenase (LDH) activities (nmol/min/mg protein) in the homogenates FS: female saline. FT: female thiamet G. MS: male saline. MT: male thiamet G. **Figure S9.** Western blot analyses of proteins related to mitochondrial function, and cellular metabolism. Western blot and quantifications of citrate synthase A), SOD2 B), HK1, Aconitase, and VDAC C), LDHA D), and glutaminase E), n = 12 each group each sex. **Figure S10.** Comparisons of mtDNA copy number and mtDNA damage between sexes and in response to thiamet G (TG). A) We used real-time PCR to measure mtDNA copy number and normalized to nuclear DNA copy number. B) To measure relative mtDNA damage, we performed PCR analyses of long 16 kb mtDNA, and a short mtDNA fragment (80 bp). Lesion frequency was calculated as described in Method, n = 12 mice each sex each treatment group. **Figure S11.** Western blot analyses of proteins related to autophagy-lysosomal degradation: LC3, P62, and LAMP1. Western blot and quantifications of LC3 A) (quantification of LC3I, and LC3II are shown), P62 B), and LAMP1 C). n = 12 each group each sex. **Figure S12.** Western blot analyses of synaptic proteins, and proteins related to Alzheimer’s and Parkinson’s diseases. Western blot and quantifications α-synuclein A), TPPP B), PSD95, PICALM, and synaptophysin C), n = 12 each group each sex.

**Additional file 2: Figure S13.** Correlation matrix heatmaps with Kendall’s rank correlation coefficients (Tau coefficients). MS: male saline, MT: male Thiamet G, FS: female saline, FT: female Thiamet G. Blue and red dots indicate negative and positive correlations, respectively. The dot sizes are proportional to the magnitudes of correlation coefficients. The color scale is shown under each heatmap. **Figure S14.** Scatter plots with regression lines for OGA activity and LAMP1 relationship. Fs: female saline, ft: female Thiamet G, ms: male saline, mt: male Thiamet G. Kendall’s correlation coefficients and p-values are shown for each group. **Figure S15.** Scatter plots with regression lines for OGA activity and LC3I relationship. Fs: female saline, ft: female Thiamet G, ms: male saline, mt: male Thiamet G. Kendall’s correlation coefficients and p-values are shown for each group. **Figure S16.** Scatter plots with regression lines for O-GlcNAc level and LC3I relationship. Fs: female saline, ft: female Thiamet G, ms: male saline, mt: male Thiamet G. Kendall’s correlation coefficients and p-values are shown for each group. **Figure S17.** Scatter plots with regression lines for LC3II and complex I (C_I) relationship. Fs: female saline, ft: female Thiamet G, ms: male saline, mt: male Thiamet G. Kendall’s correlation coefficients and p-values are shown for each group. **Figure S18.** Scatter plots with regression lines for PICALM and complex III (C_III) relationship. Fs: female saline, ft: female Thiamet G, ms: male saline, mt: male Thiamet G. Kendall’s correlation coefficients and p-values are shown for each group.

**Additional file 3:** Supplementary Tables S1 to S6.

## Data Availability

Please contact author for data requests.
