# Supplementary material for: Acute inhibition of OGA sex-dependently alters the networks associated with bioenergetics, autophagy, and neurodegeneration
Source: Mol Brain. 2022 Mar 5;15:22. doi: 10.1186/s13041-022-00906-x (PMC8898497; doi:10.1186/s13041-022-00906-x)
Supplement: Supplementary file 1 — Additional file 1: Figure S1. Higher magnification images of CTD antibody immunostaining demonstrating that overall protein O-GlcNAcylation is increased in the cortex exposed to Thiamet G (TG). No primary antibody was used as a negative control. Representative images mouse from saline and TG group were shown (n = 3 each). Scale bar = 100 µm. Blue: nuclear staining. Green: CTD staining. Merge: both nuclear and CTD staining. Figure S2. Higher magnification images of CTD antibody immunostaining demonstrating that overall protein O-GlcNAcylation is increased in the hippocampus exposed to Thiamet G (TG). No primary antibody was used as a negative control. Representative images mouse from saline and TG group were shown (n = 3 each). Scale bar = 100 µm. Blue: nuclear staining. Green: CTD staining. Merge: both nuclear and CTD staining. Figure S3. Higher magnification images of anti-OGT antibody immunostaining in the cortex. No primary antibody was used as a negative control. Representative images mouse from saline and Thiamet G (TG) group were shown (n = 3 each). Scale bar = 100 µm. Blue: nuclear staining. Red: OGT staining. Merge: both nuclear and OGT staining. Figure S4. Higher magnification images of anti-OGT antibody immunostaining in the hippocampus. No primary antibody was used as a negative control. Representative images mouse from saline and Thiamet G (TG) group were shown (n = 3 each). Scale bar = 100 µm. Blue: nuclear staining. Red: OGT staining. Merge: both nuclear and OGT staining. Figure S5. Higher magnification images of anti-OGA antibody immunostaining in the cortex. No primary antibody was used as a negative control. Representative images mouse from saline and Thiamet G (TG) group were shown (n = 3 each). Scale bar = 100 µm. Blue: nuclear staining. Red: OGA staining. Merge: both nuclear and OGA staining. Figure S6. Higher magnification images of anti-OGA antibody immunostaining in the hippocampus. No primary antibody was used as a negative control. Represent [file 13041_2022_906_MOESM1_ESM.pptx]

## Slide 1
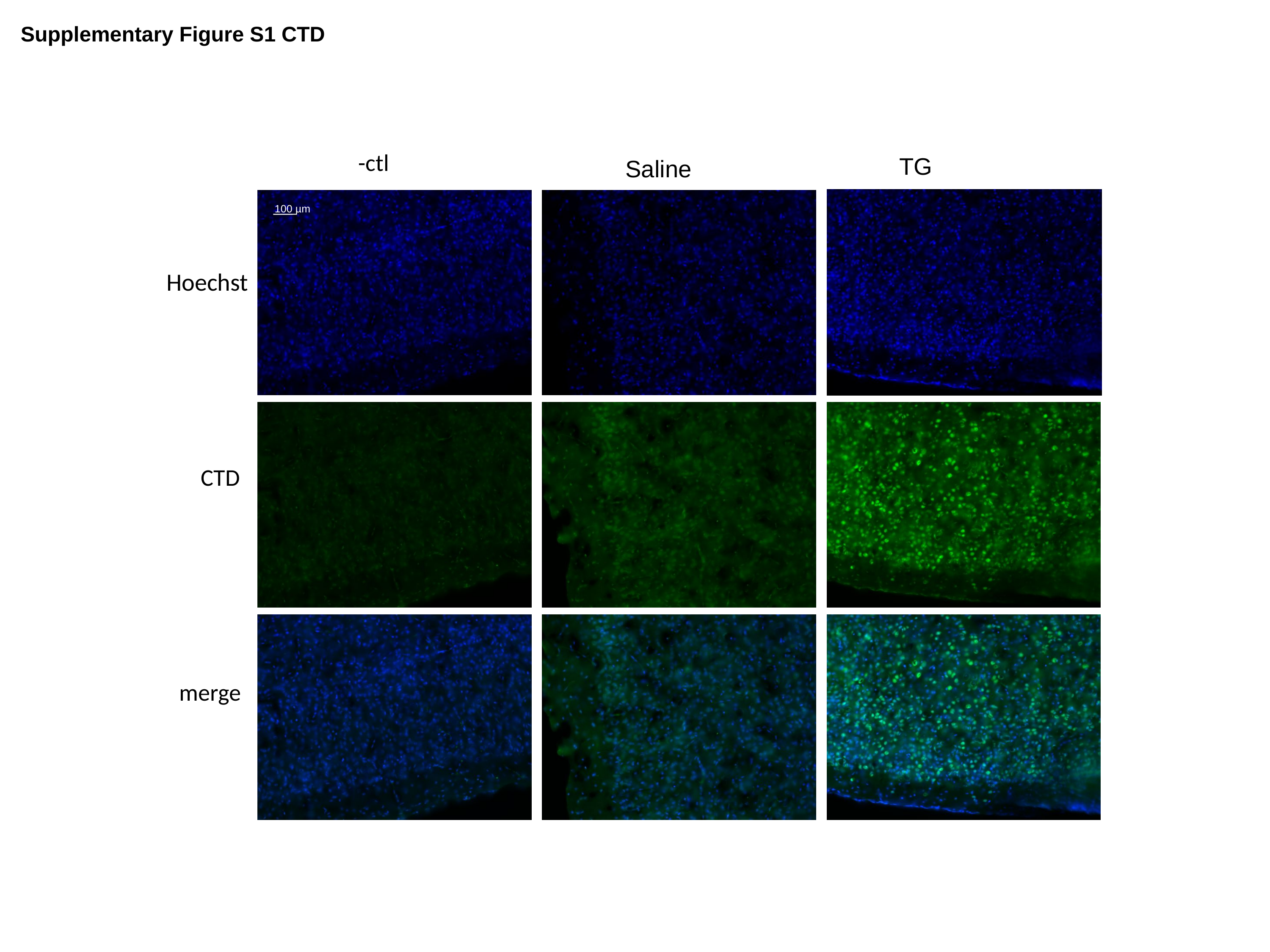

Supplementary Figure S1 CTD
-ctl
TG
Saline
100 µm
Hoechst
CTD
merge

## Slide 2
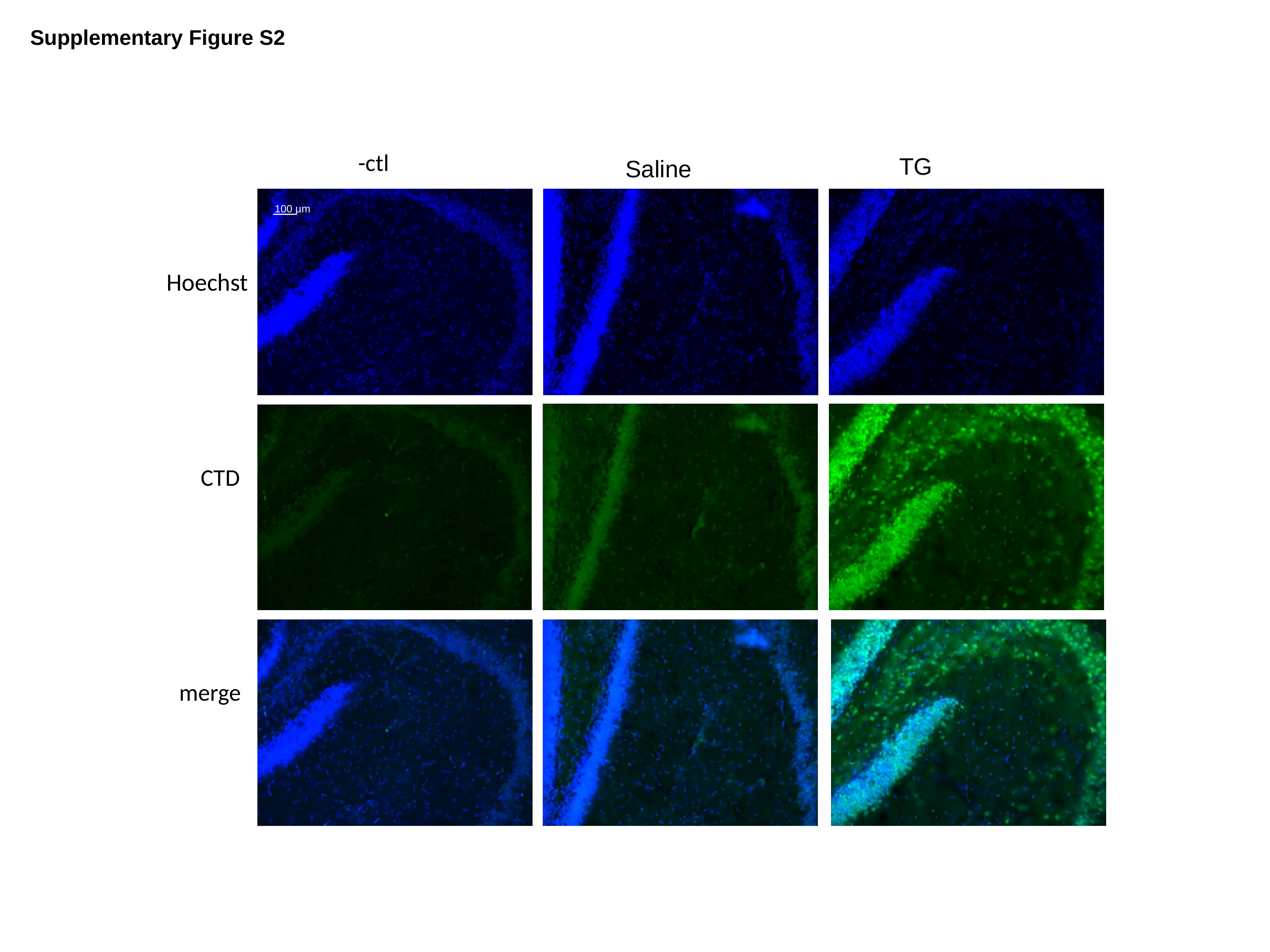

Supplementary Figure S2
-ctl
TG
Saline
100 µm
Hoechst
CTD
merge

## Slide 3
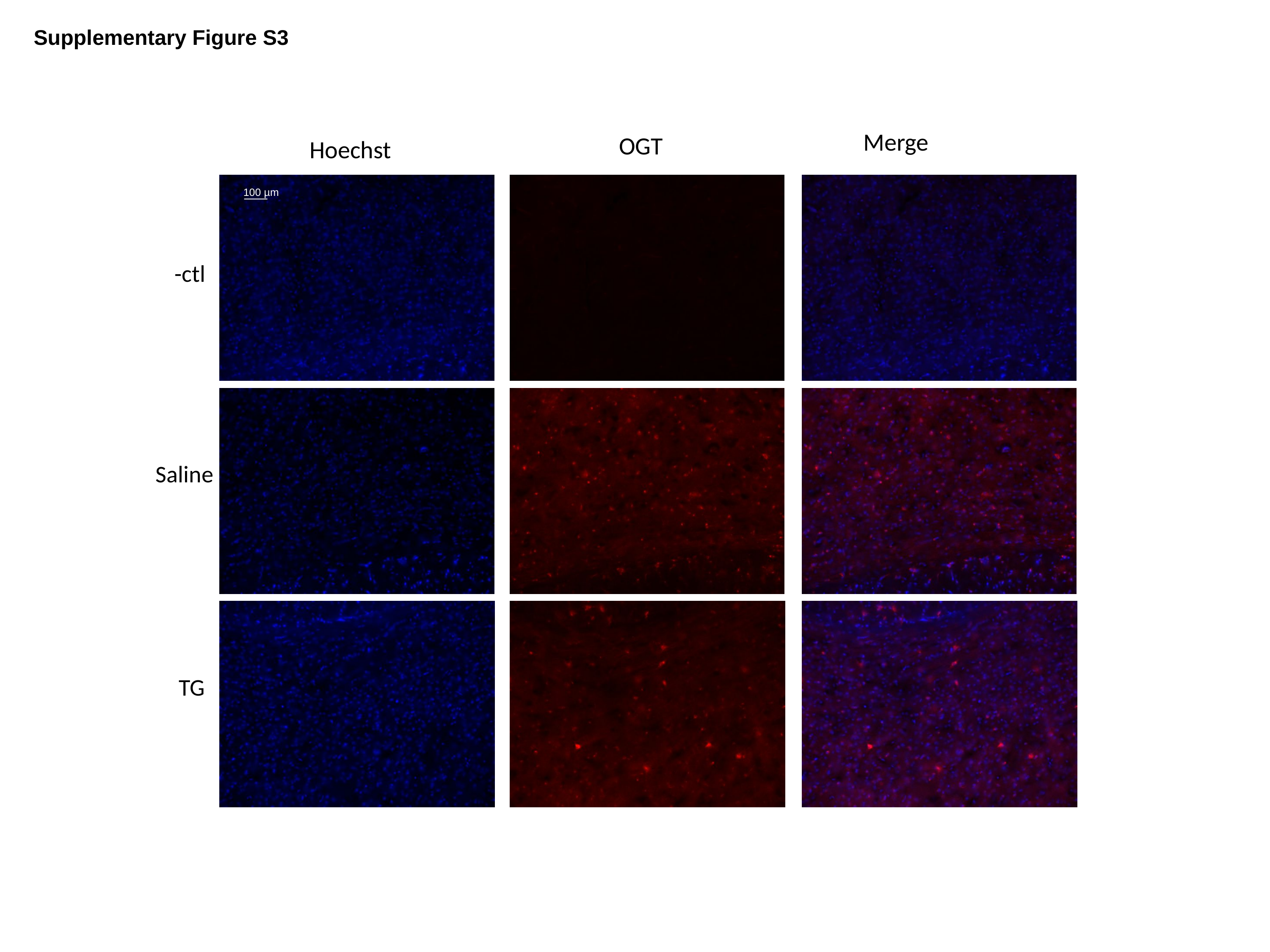

Supplementary Figure S3
Merge
OGT
Hoechst
100 µm
-ctl
Saline
TG

## Slide 4
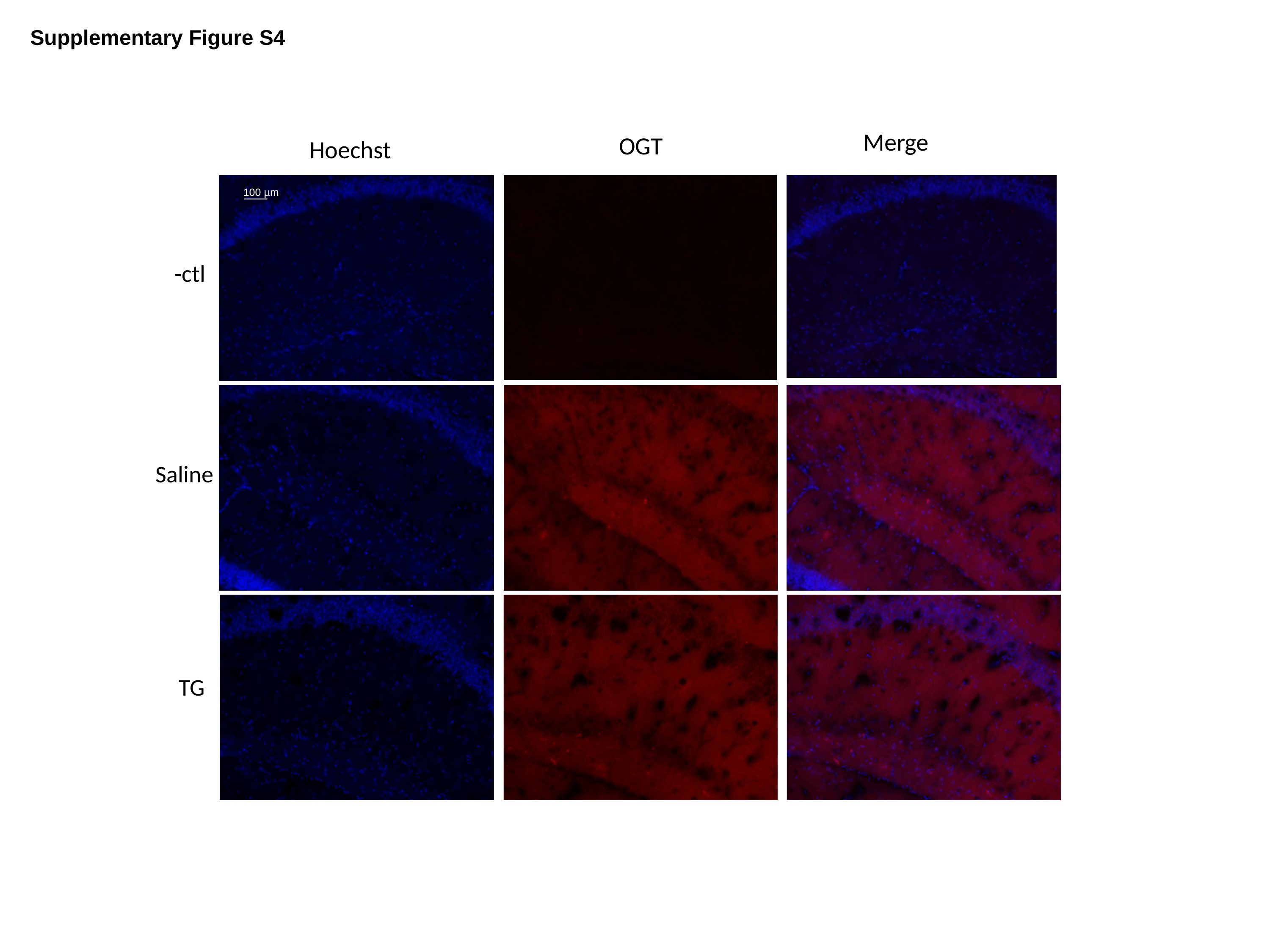

Supplementary Figure S4
Merge
OGT
Hoechst
100 µm
-ctl
Saline
TG

## Slide 5
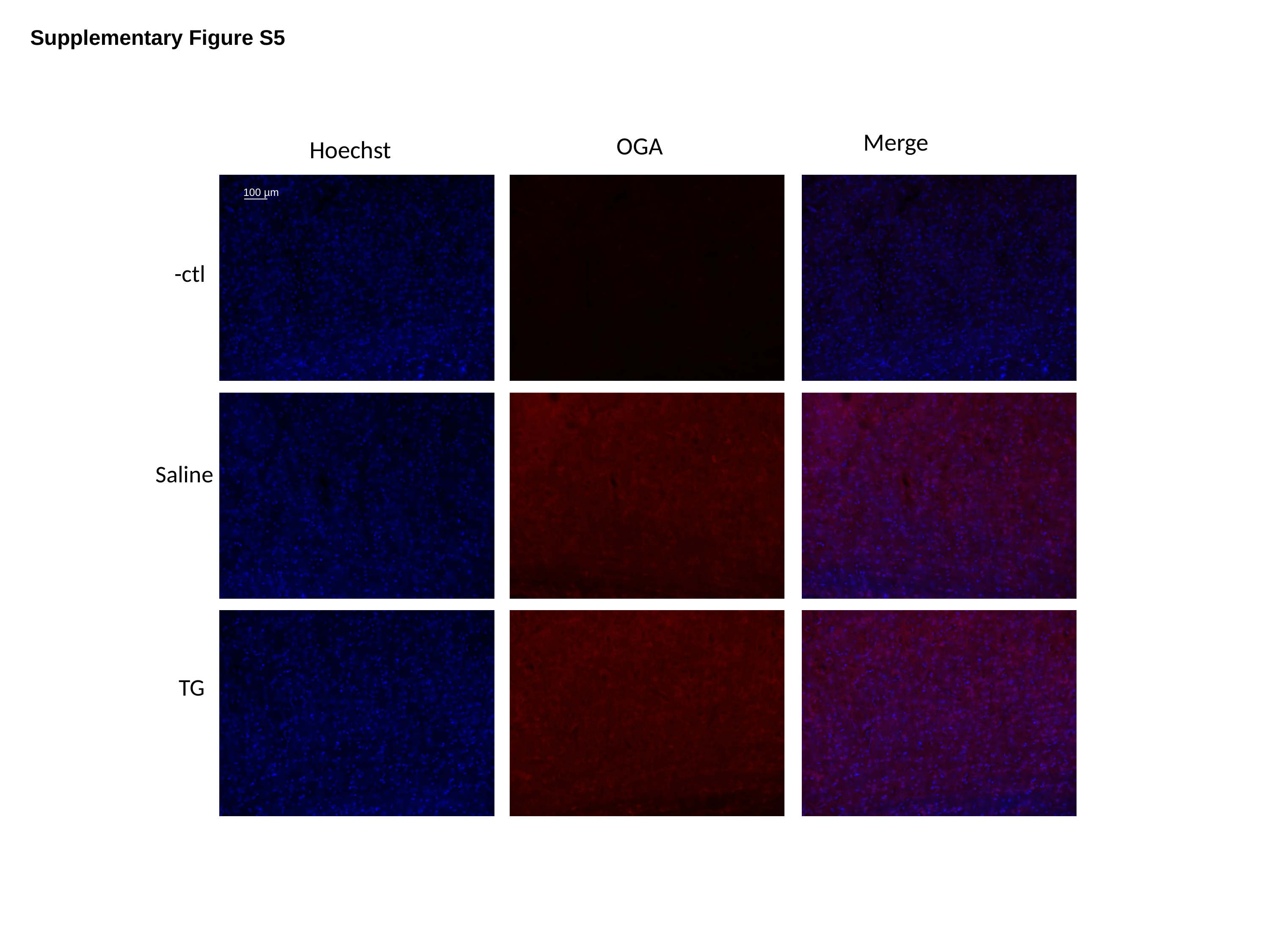

Supplementary Figure S5
Merge
OGA
Hoechst
100 µm
-ctl
Saline
TG

## Slide 6
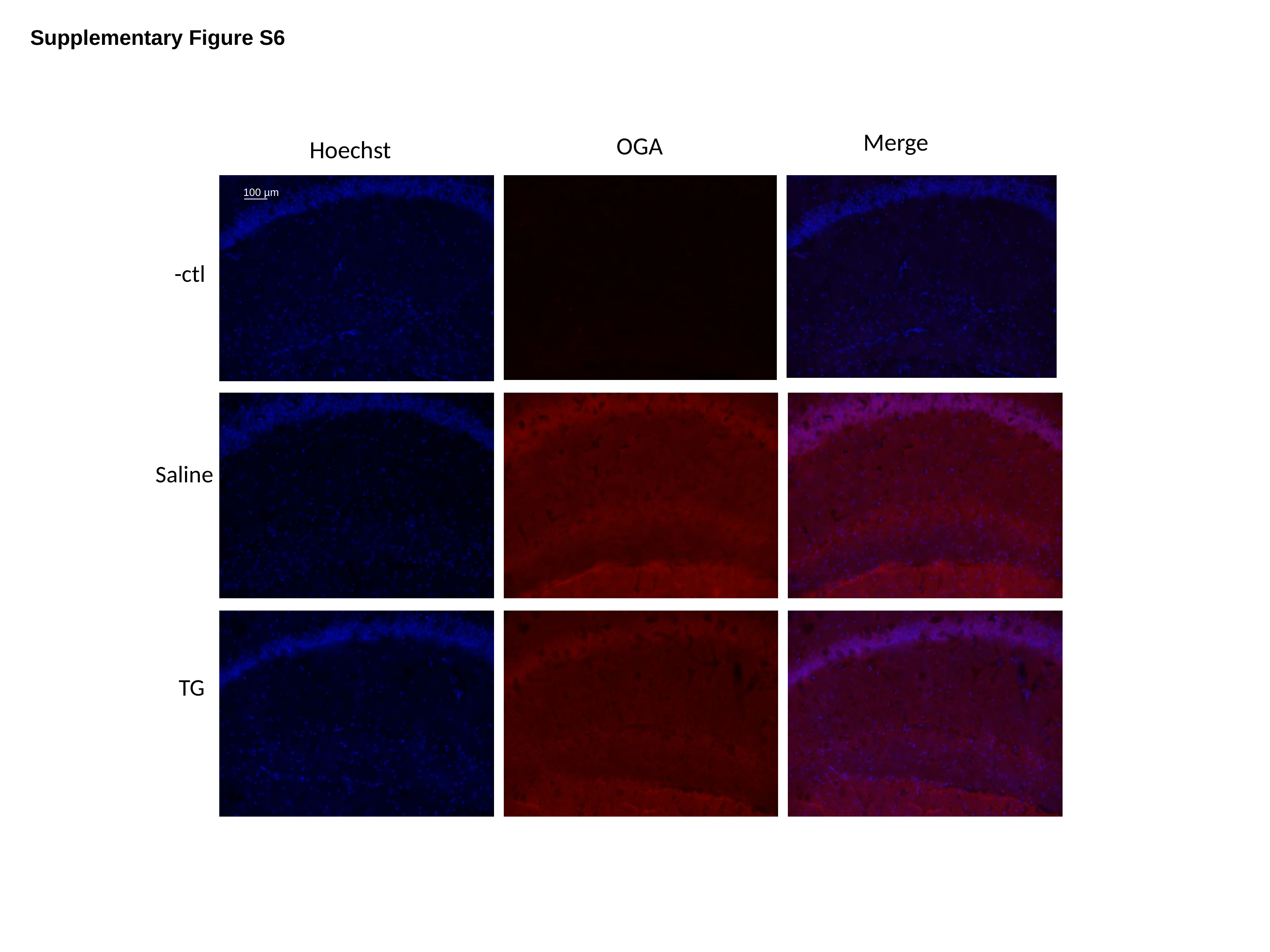

Supplementary Figure S6
Merge
OGA
Hoechst
100 µm
-ctl
Saline
TG

## Slide 7
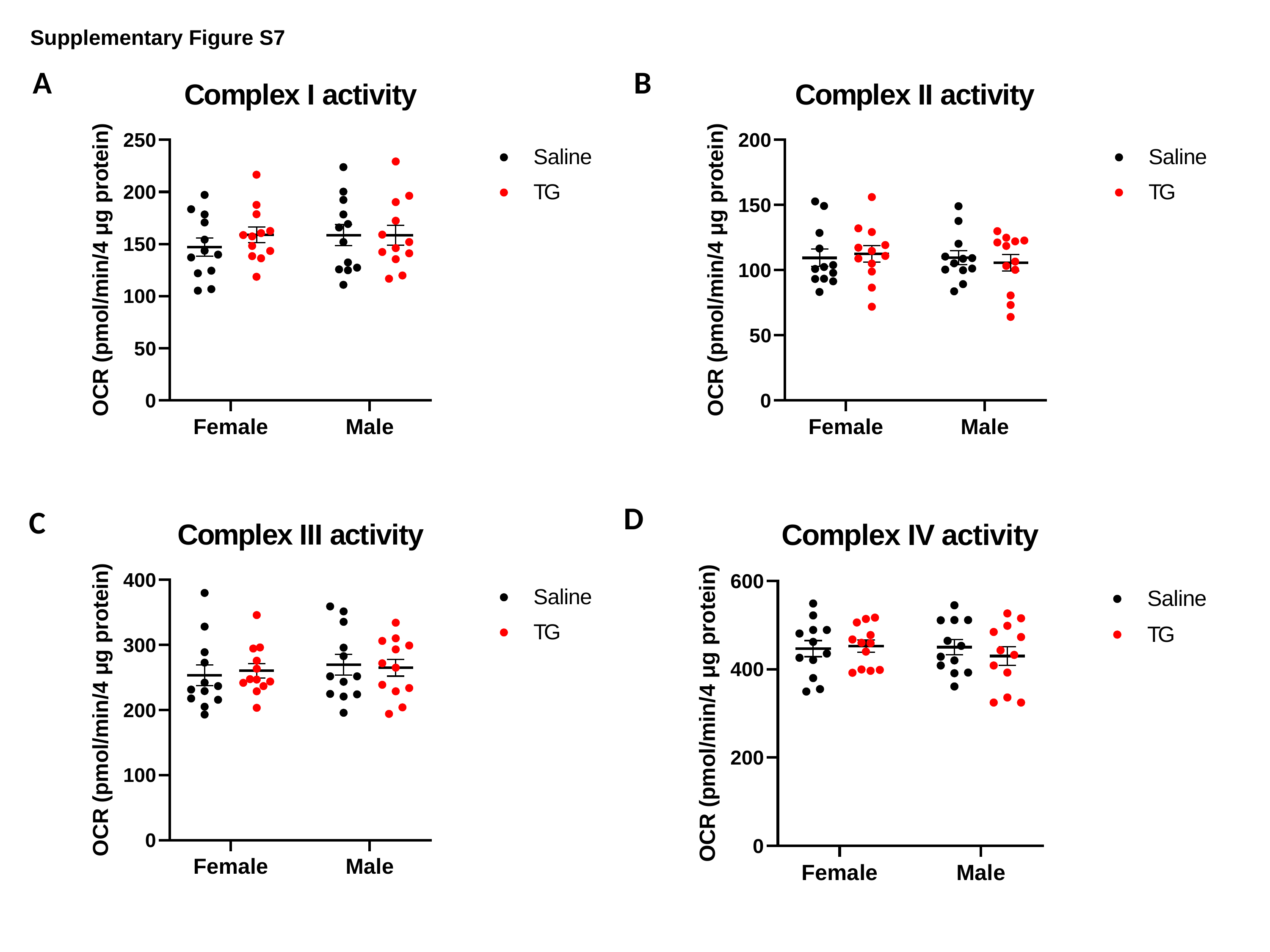

Supplementary Figure S7
A
B
D
C

## Slide 8
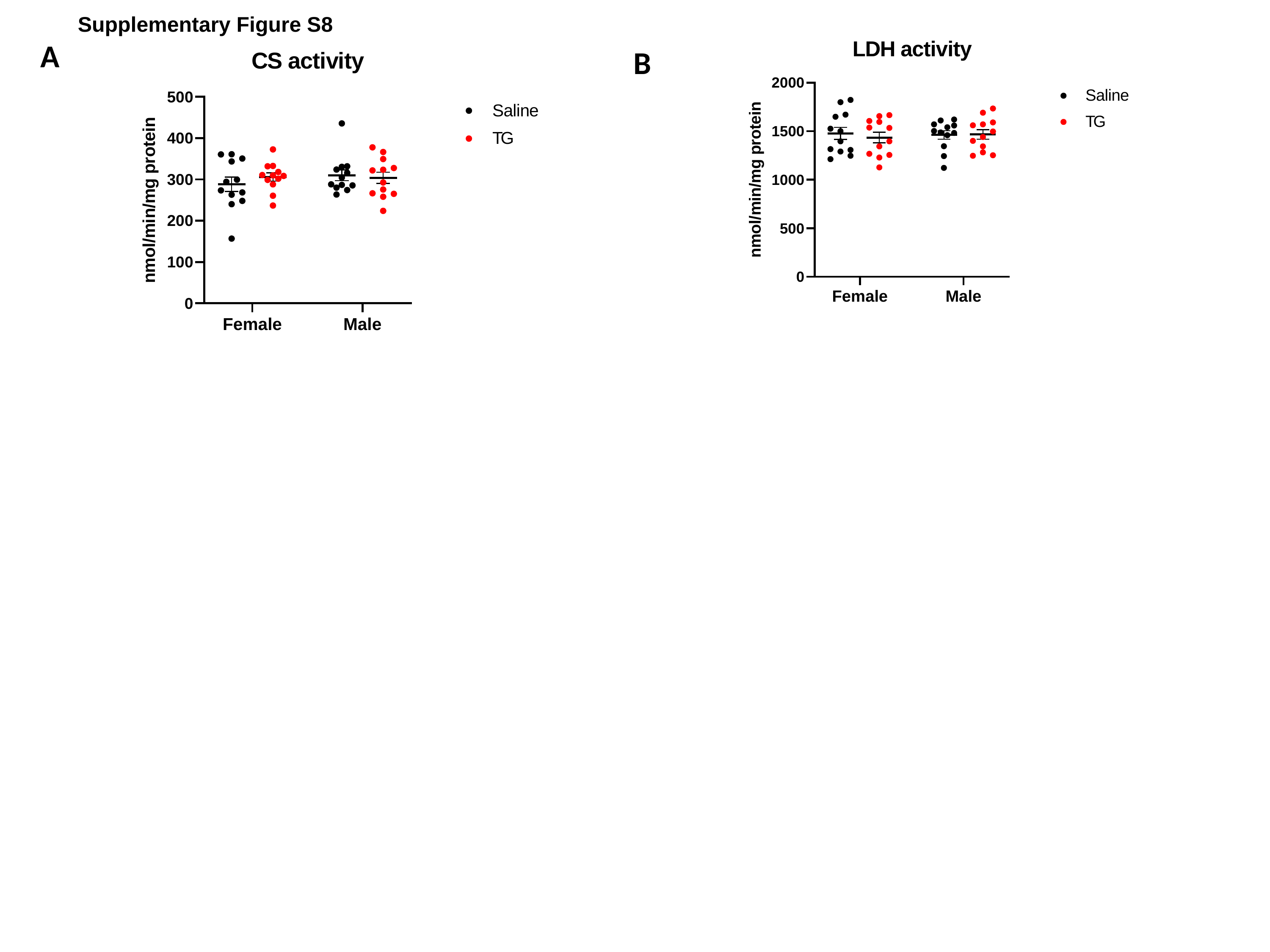

Supplementary Figure S8
A
B

## Slide 9
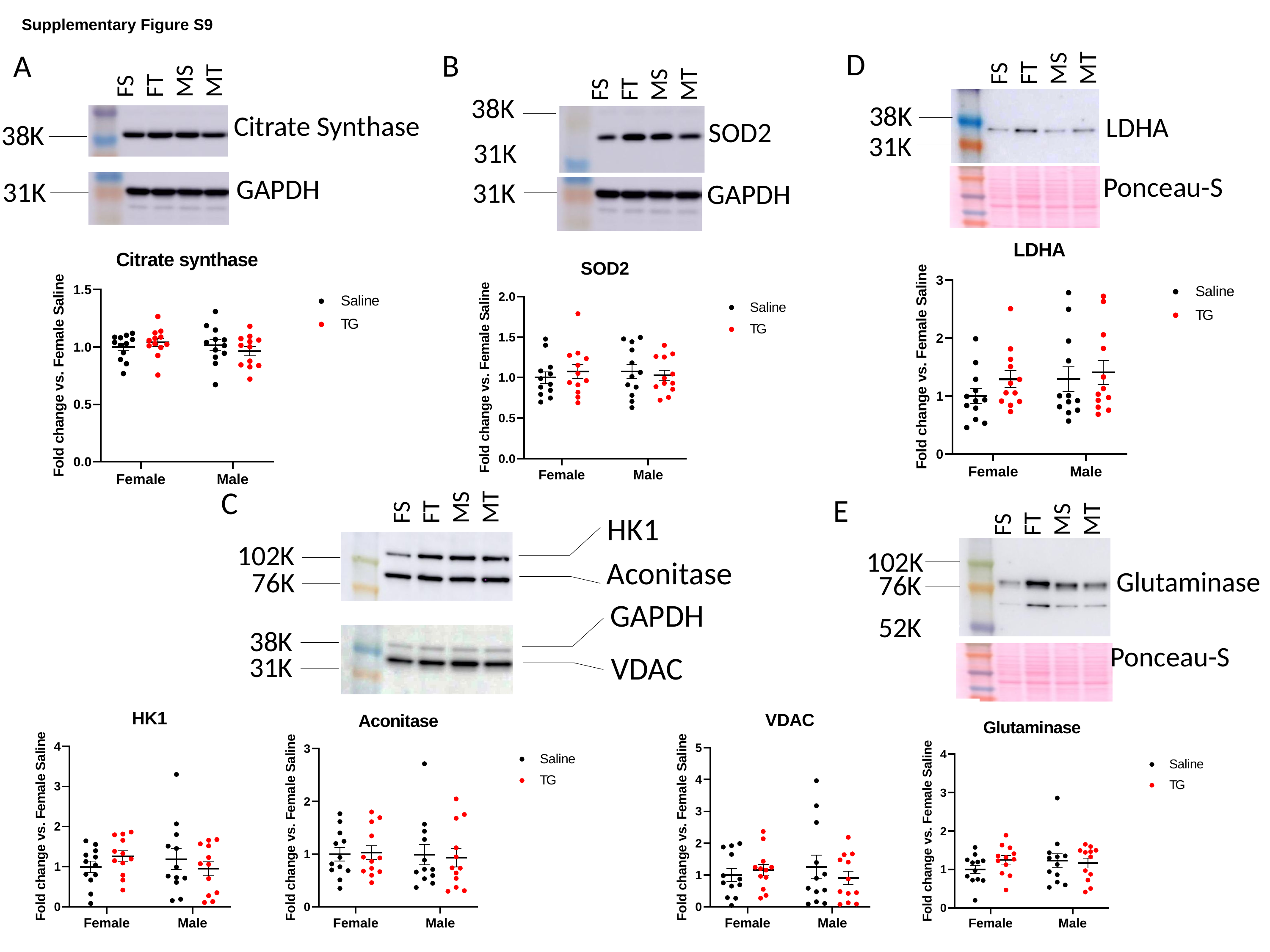

FS
FT
MS
MT
Supplementary Figure S9
FS
FT
MS
MT
FS
FT
MS
MT
D
B
A
38K
SOD2
31K
31K
GAPDH
38K
LDHA
31K
Ponceau-S
Citrate Synthase
38K
GAPDH
31K
FS
FT
MS
MT
FS
FT
MS
MT
C
E
HK1
102K
102K
Glutaminase
76K
52K
Ponceau-S
Aconitase
76K
GAPDH
38K
VDAC
31K

## Slide 10
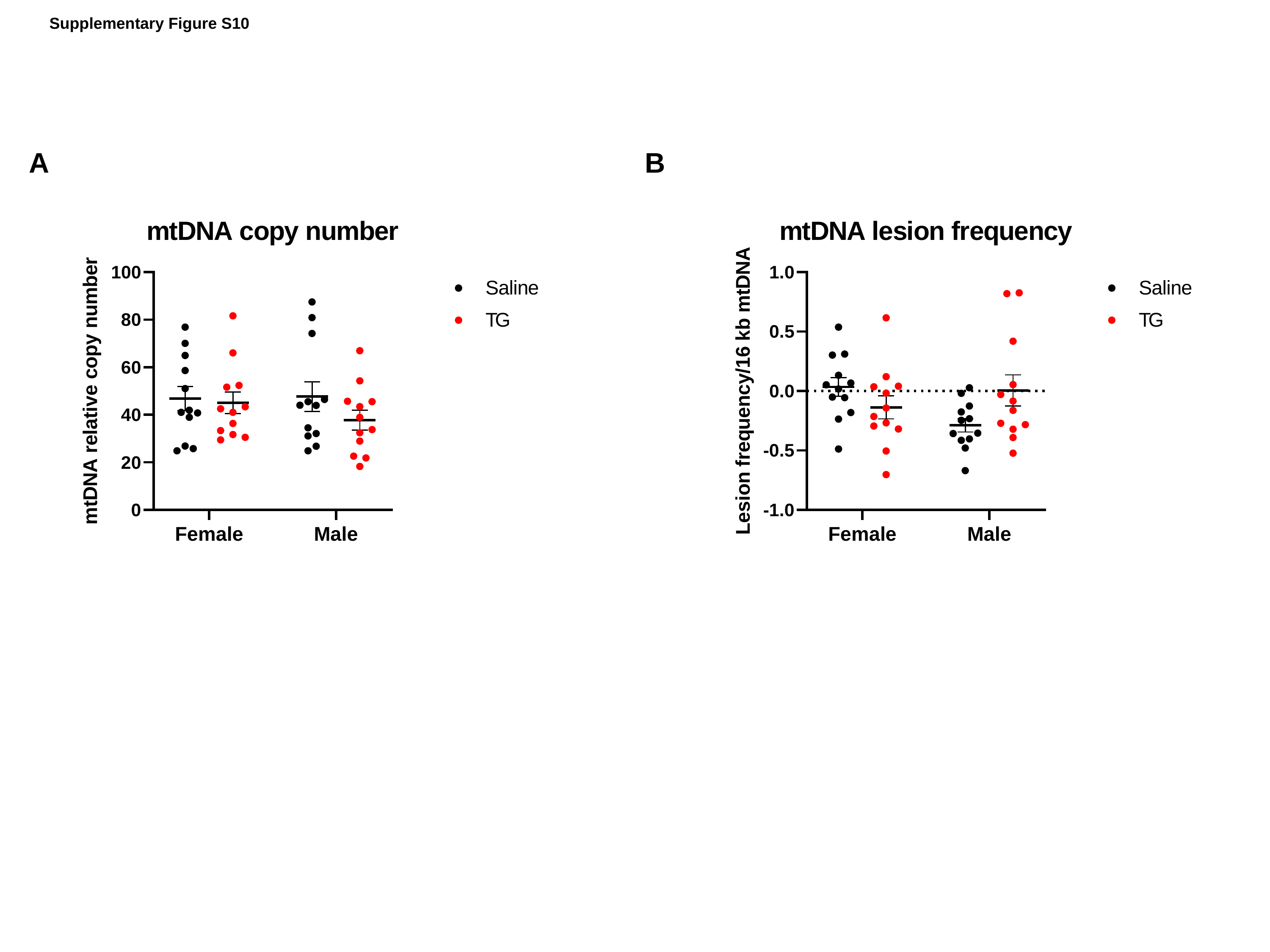

Supplementary Figure S10
# A
B

## Slide 11
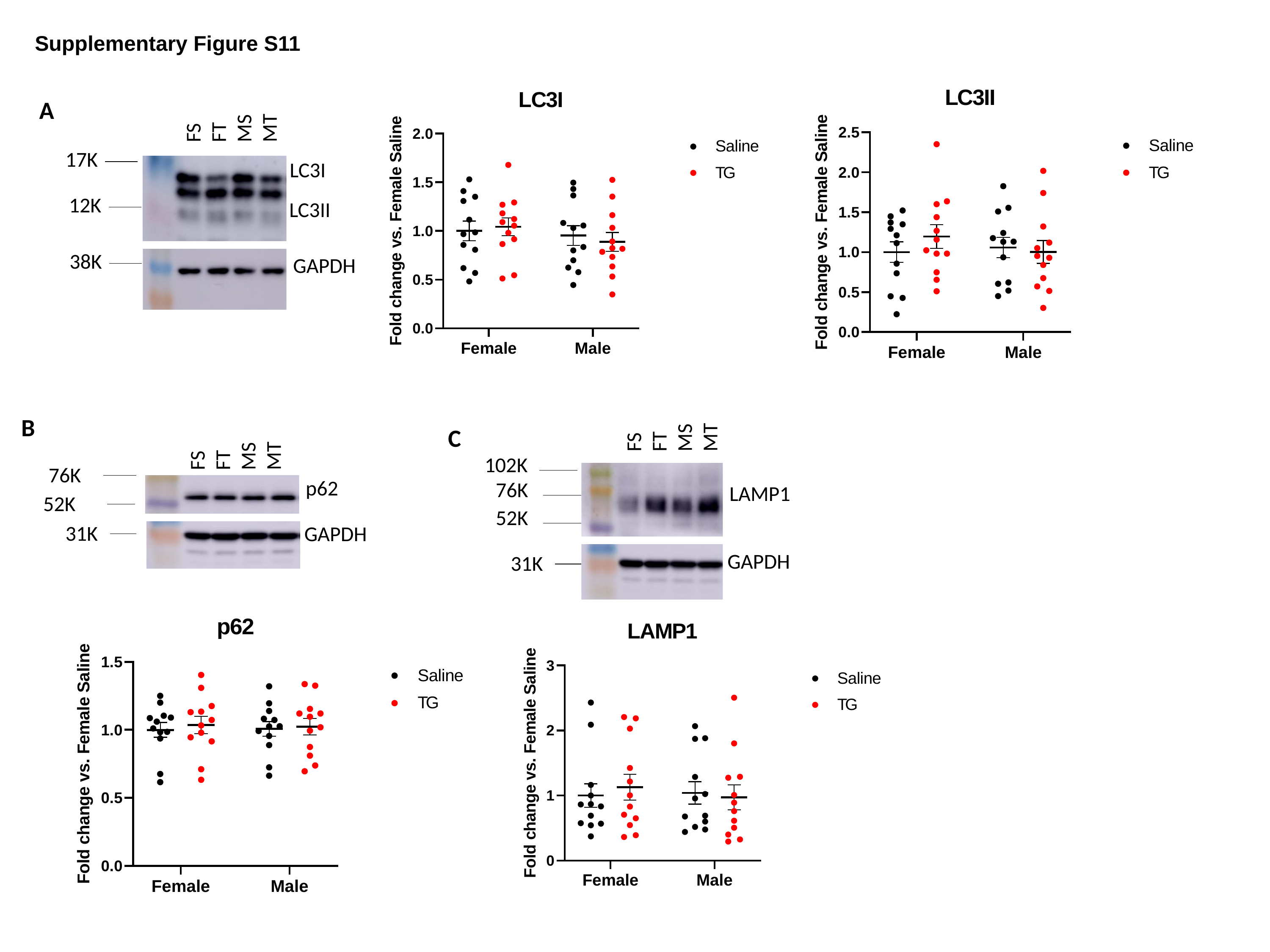

Supplementary Figure S11
FS
FT
MS
MT
A
17K
LC3I
12K
LC3II
38K
GAPDH
FS
FT
MS
MT
B
FS
FT
MS
MT
102K
76K
52K
C
76K
p62
LAMP1
52K
31K
GAPDH
GAPDH
31K

## Slide 12
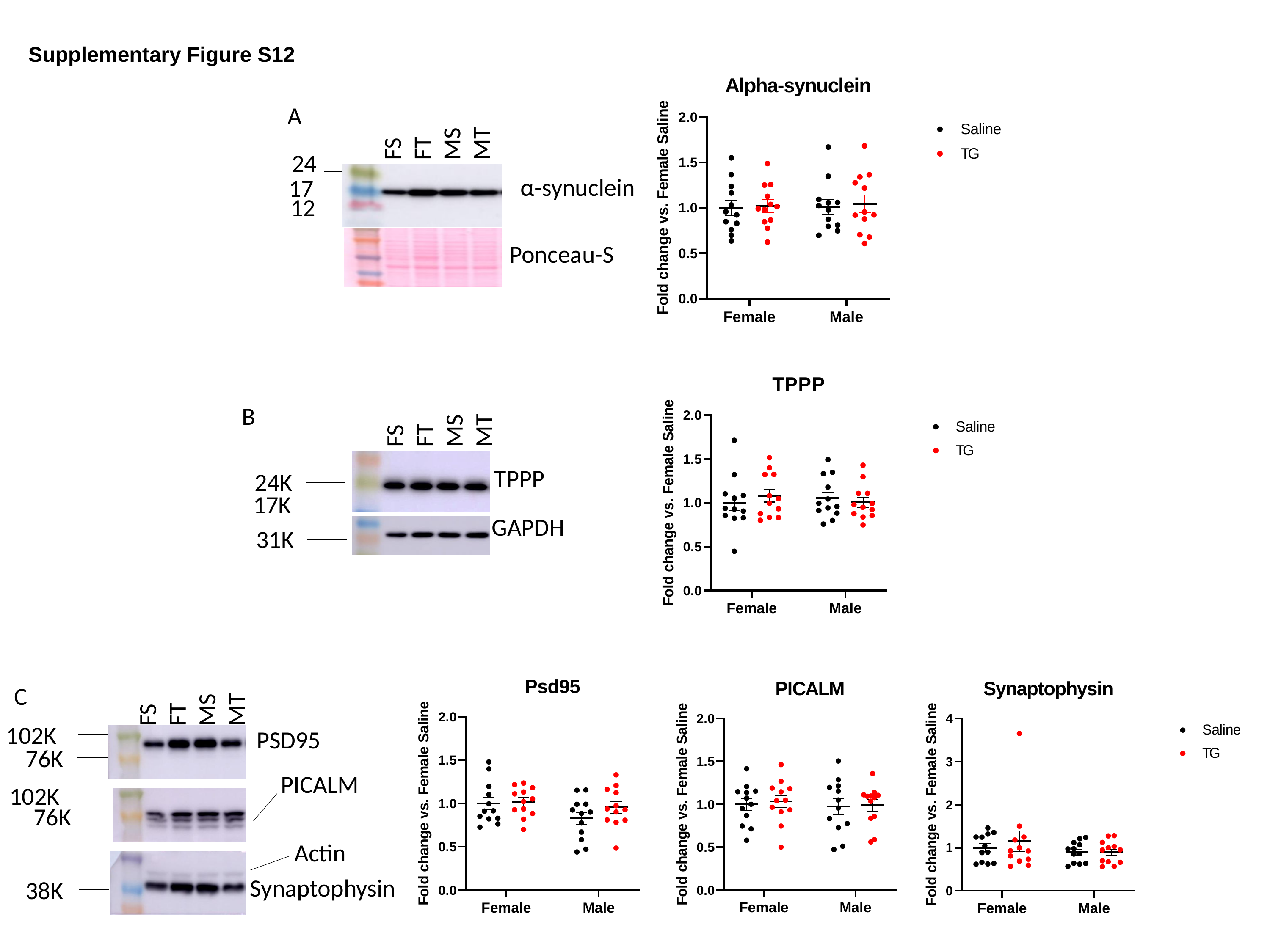

Supplementary Figure S12
FS
FT
MS
MT
A
24
α-synuclein
17
12
Ponceau-S
FS
FT
MS
MT
B
TPPP
24K
17K
GAPDH
31K
FS
FT
MS
MT
C
102K
PSD95
76K
PICALM
102K
76K
Actin
Synaptophysin
38K
