# Supplementary material for: Acute inhibition of OGA sex-dependently alters the networks associated with bioenergetics, autophagy, and neurodegeneration
Source: Mol Brain. 2022 Mar 5;15:22. doi: 10.1186/s13041-022-00906-x (PMC8898497; doi:10.1186/s13041-022-00906-x)
Supplement: Supplementary file 2 — Additional file 2: Figure S13. Correlation matrix heatmaps with Kendall’s rank correlation coefficients (Tau coefficients). MS: male saline, MT: male Thiamet G, FS: female saline, FT: female Thiamet G. Blue and red dots indicate negative and positive correlations, respectively. The dot sizes are proportional to the magnitudes of correlation coefficients. The color scale is shown under each heatmap. Figure S14. Scatter plots with regression lines for OGA activity and LAMP1 relationship. Fs: female saline, ft: female Thiamet G, ms: male saline, mt: male Thiamet G. Kendall’s correlation coefficients and p-values are shown for each group. Figure S15. Scatter plots with regression lines for OGA activity and LC3I relationship. Fs: female saline, ft: female Thiamet G, ms: male saline, mt: male Thiamet G. Kendall’s correlation coefficients and p-values are shown for each group. Figure S16. Scatter plots with regression lines for O-GlcNAc level and LC3I relationship. Fs: female saline, ft: female Thiamet G, ms: male saline, mt: male Thiamet G. Kendall’s correlation coefficients and p-values are shown for each group. Figure S17. Scatter plots with regression lines for LC3II and complex I (C_I) relationship. Fs: female saline, ft: female Thiamet G, ms: male saline, mt: male Thiamet G. Kendall’s correlation coefficients and p-values are shown for each group. Figure S18. Scatter plots with regression lines for PICALM and complex III (C_III) relationship. Fs: female saline, ft: female Thiamet G, ms: male saline, mt: male Thiamet G. Kendall’s correlation coefficients and p-values are shown for each group. [file 13041_2022_906_MOESM2_ESM.pptx]

## Slide 1
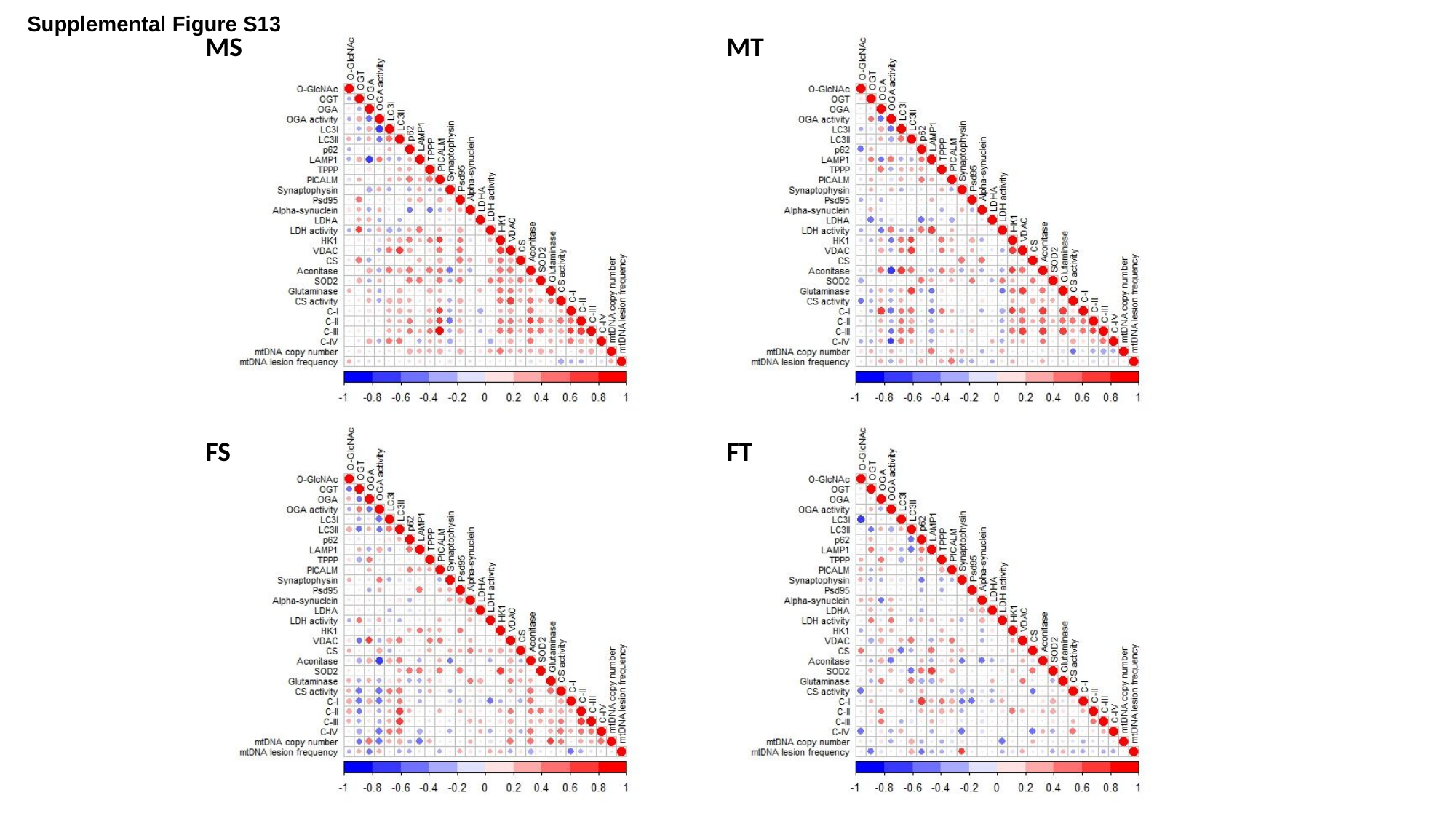

Supplemental Figure S13
MS
MT
FS
FT

## Slide 2
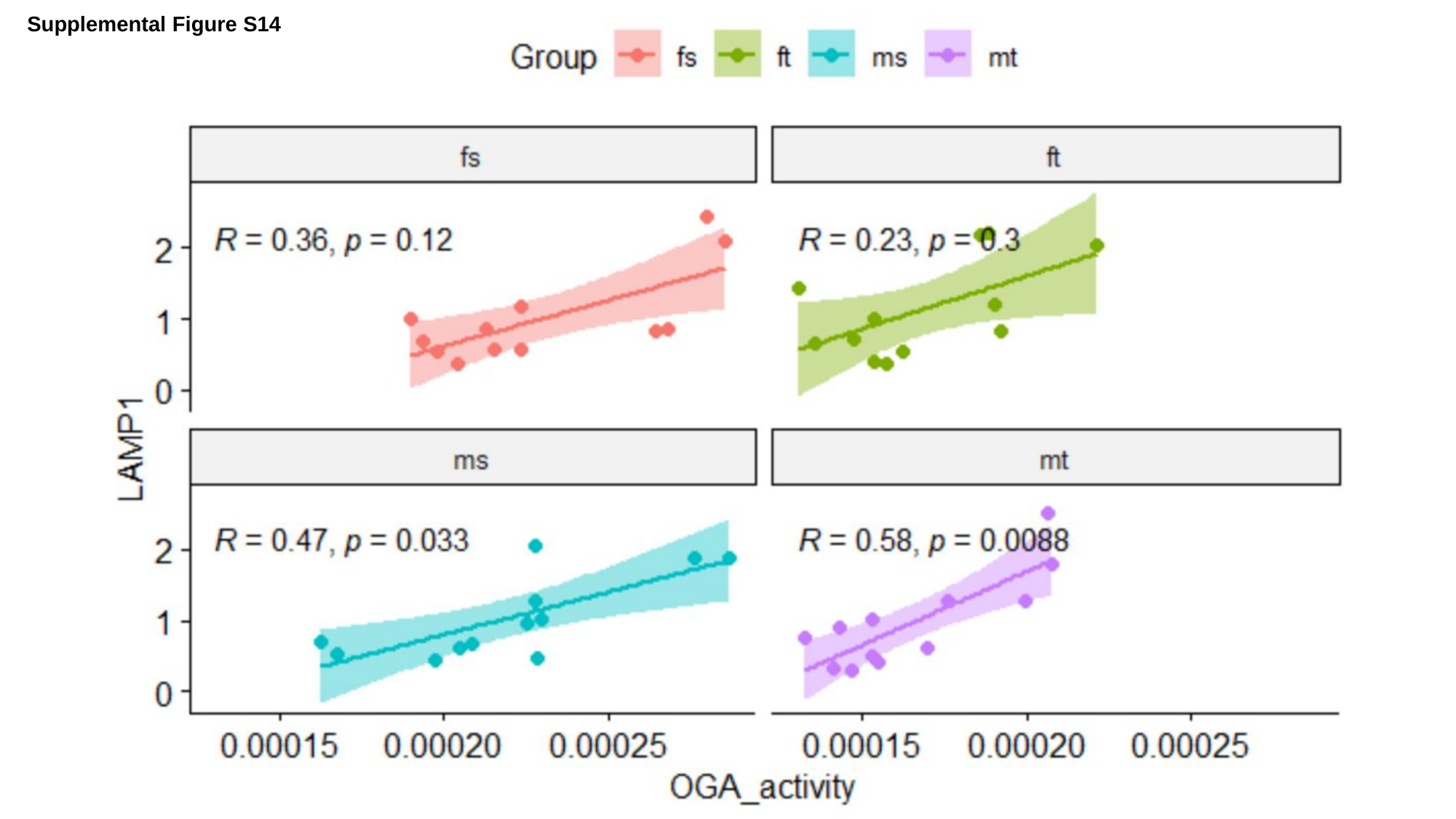

Supplemental Figure S14

## Slide 3
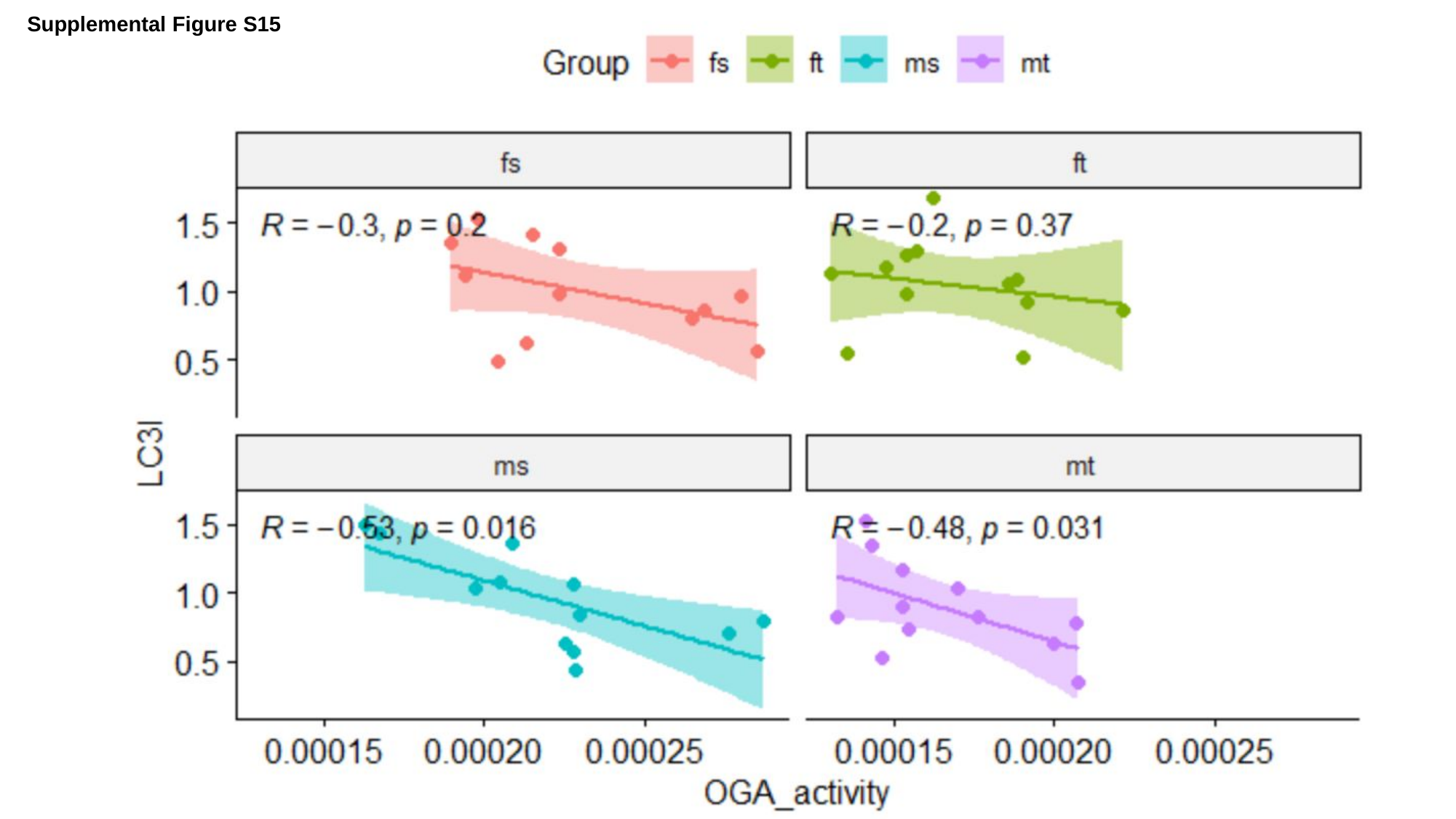

Supplemental Figure S15

## Slide 4
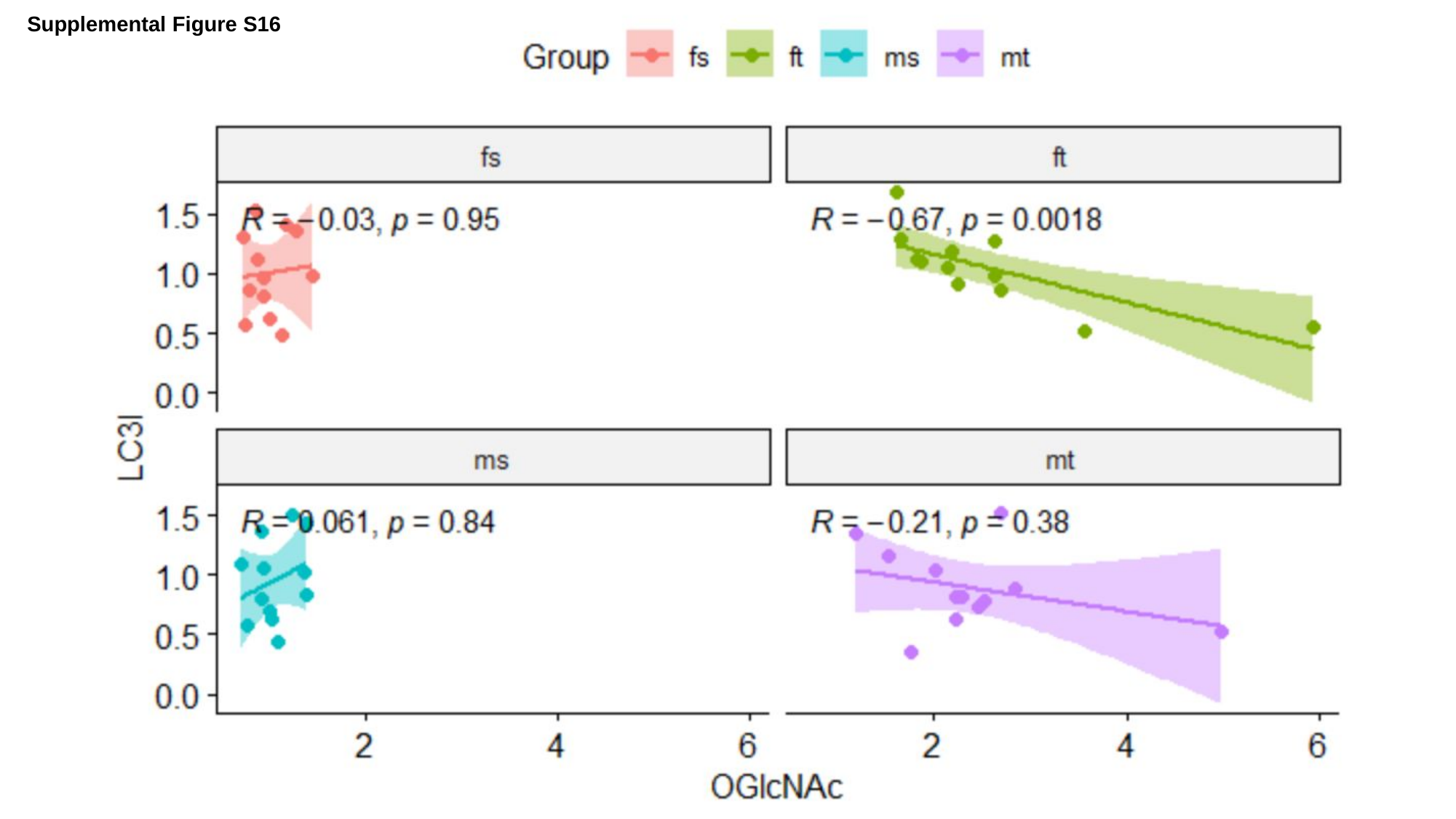

Supplemental Figure S16
#

## Slide 5
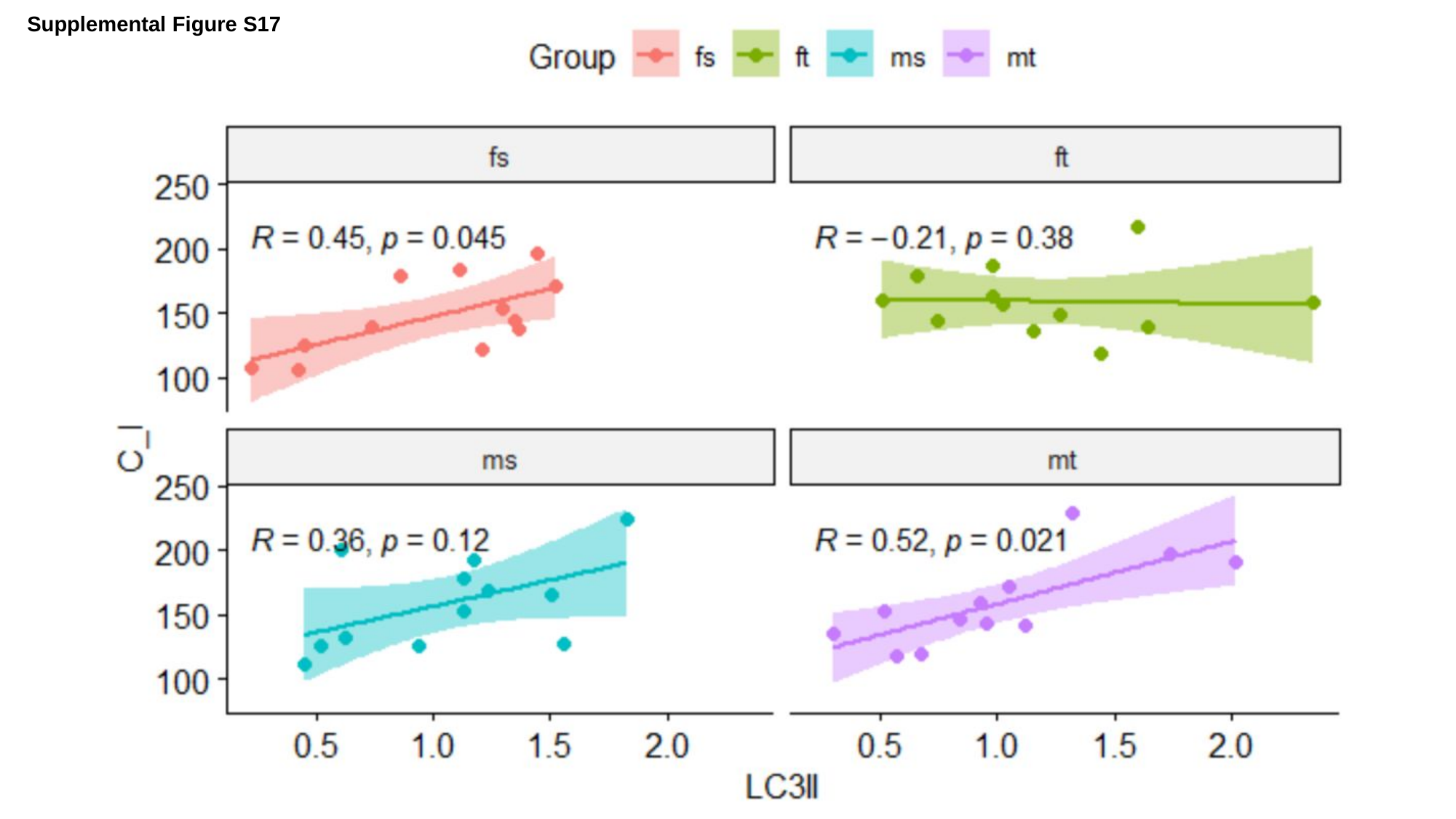

Supplemental Figure S17
#

## Slide 6
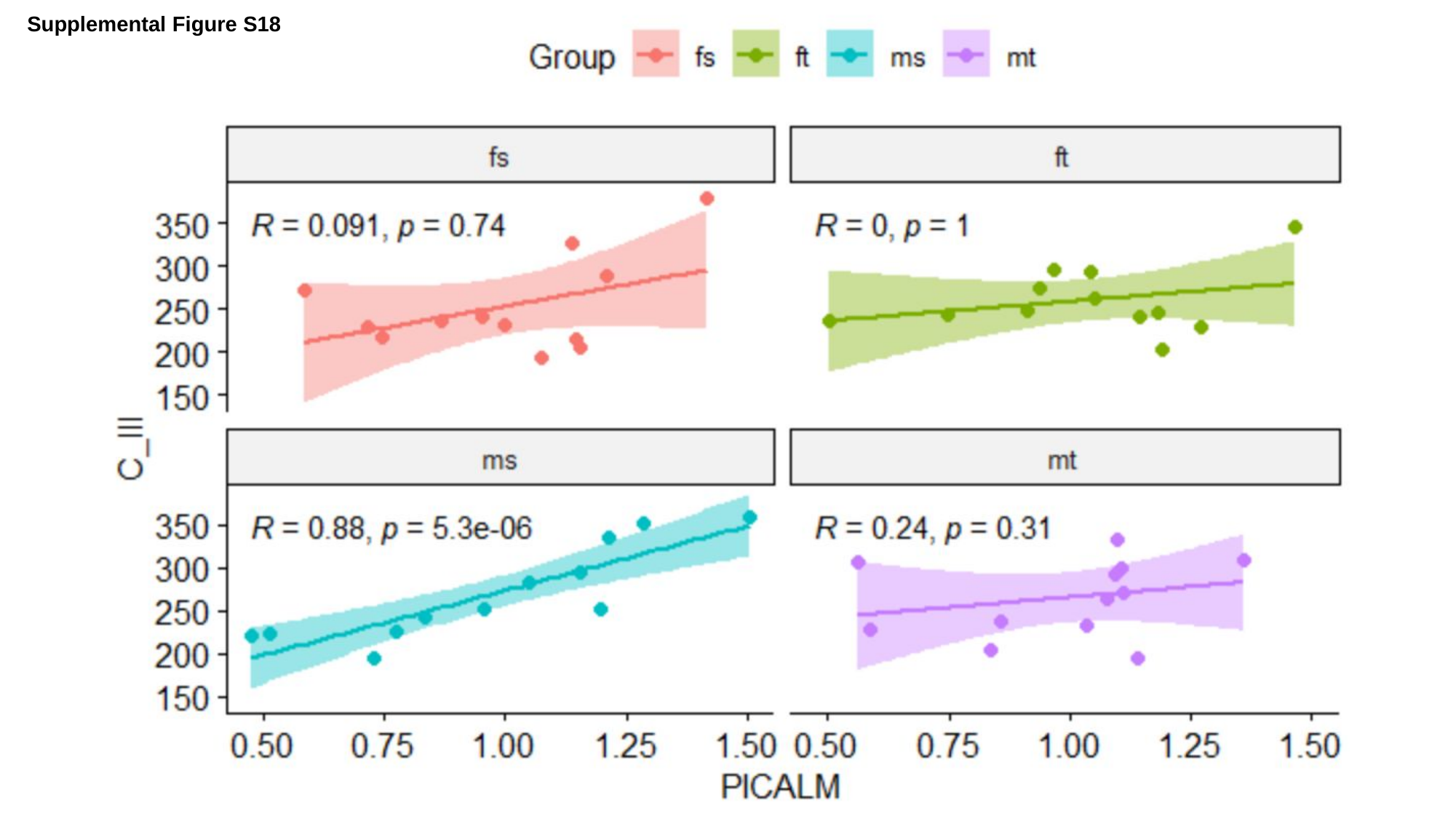

Supplemental Figure S18
#
